# Supplementary material for: From short to long term: Dynamic analysis of FDI and net export in global regions
Source: PLoS One. 2023 Sep 14;18(9):e0291301. doi: 10.1371/journal.pone.0291301 (PMC10501631; doi:10.1371/journal.pone.0291301)
Supplement: S1 Appendix — (DOCX) [file pone.0291301.s001.docx]

# Appendix S1. Summary of the Continents and the Countries

| **Continents** | **Countries** |
| --- | --- |
| Africa | Angola, Botswana, Cabo Verde, Djibouti, Egypt Arab Rep., Eswatini (Swaziland), Ethiopia, Ghana, Kenya, Madagascar, Malawi, Mauritius, Morocco, Namibia, Nigeria, Sao Tome and Principe, Seychelles, Sierra Leone, South Africa, Tunisia, Zambia |
| Asia and Oceanian | Armenia, Azerbaijan, Bangladesh, Cambodia, China, India, Indonesia, Israel, Japan, Kazakhstan, Korea Rep, Kuwait, Kyrgyz Republic, Malaysia, Maldives, Mongolia, Nepal, Oman, Pakistan, Philippines, Saudi Arabia, Singapore, Sri Lanka, Thailand, Vietnam |
|  | Australia, Solomon Islands, Vanuatu |
| Europe | Albania, Belarus, Bulgaria, Croatia, Cyprus, Czech Republic, Denmark, Estonia, Finland, France, Germany, Greece, Hungary, Iceland, Italy, Latvia, Lithuania, Malta, Moldova, Netherlands, North Macedonia, Norway, Poland, Portugal, Romania, Russian Federation, Slovak Republic, Slovenia, Spain, Sweden, Switzerland, Turkey, Ukraine, United Kingdom |
| North America | Antigua and Barbuda, Bahamas, Belize, Canada, Costa Rica, Dominica, Dominican Republic, Grenada, Guatemala, Jamaica, Mexico, Nicaragua, Panama, St. Lucia, St. Vincent and the Grenadines, Trinidad and Tobago, United States |
| South America | Argentina, Bolivia, Brazil, Chile, Colombia, Ecuador, Guyana, Paraguay, Peru, Uruguay |

Source: Authors’ compilation based on [World Population Review (2022)](file:///C:\Users\sande\Downloads\Group%20No.24%20(2).docx#_ENREF_58)
