# Supplementary material for: From short to long term: Dynamic analysis of FDI and net export in global regions
Source: PLoS One. 2023 Sep 14;18(9):e0291301. doi: 10.1371/journal.pone.0291301 (PMC10501631; doi:10.1371/journal.pone.0291301)
Supplement: S3 Appendix — (DOCX) [file pone.0291301.s003.docx]

# Appendix S3. Unit root test results

### Table 01: Unit root test results - European Countries

| **Country** | **Augmented Dickey–Fuller** | | **Phillips–Perron** | |
| --- | --- | --- | --- | --- |
|  | **FDI** | **NE** | **FDI** | **NE** |
| Albania | -3.181**^D1^ | -3.966*** ^D1^ | -3.134** ^D1^ | -3.964*** ^D1^ |
| Belarus | -2.729* | -4.419*** ^D1^ | -2.659* | -4.522*** ^D1^ |
| Bulgaria | -2.704* ^D1^ | -3.225** ^D1^ | -2.668* ^D1^ | -3.207** ^D1^ |
| Croatia | -4.539*** ^D1^ | -3.362** ^D1^ | -4.547*** ^D1^ | -3.373** ^D1^ |
| Cyprus | -2.805* | -5.509*** ^D1^ | -2.782* | -5.398*** ^D1^ |
| Czech Republic | -4.928*** | -5.722*** ^D1^ | -4.951*** | -6.493*** ^D1^ |
| Denmark | -6.401*** | -4.717*** ^D1^ | -6.266*** | -4.804*** ^D1^ |
| Estonia | -2.928** | -3.070** ^D1^ | -2.932** | -3.042** ^D1^ |
| Finland | -5.690*** | -4.310*** ^D1^ | -5.602*** | -4.306*** ^D1^ |
| France | -4.330*** ^D1^ | -4.012*** ^D1^ | -4.362*** ^D1^ | -4.016*** ^D1^ |
| Germany | -3.006** | -3.723*** ^D1^ | -2.989** | -3.704*** ^D1^ |
| Greece | -3.788*** | -2.617* ^D1^ | -3.789*** | -2.610* ^D1^ |
| Hungary | -3.853*** ^D1^ | -2.893** ^D1^ | -3.838*** ^D1^ | -2.967** ^D1^ |
| Iceland | -4.914*** ^D1^ | -3.293**^D2^ | -5.117*** ^D1^ | -3.238** ^D2^ |
| Italy | -3.422** | -3.076** ^D1^ | -3.360** | -3.051**^D1^ |
| Latvia | -3.005** | -2.906** ^D1^ | -2.950** | -2.833* ^D1^ |
| Lithuania | -3.740*** ^D1^ | -3.352** ^D1^ | -3.719*** ^D1^ | -3.273** ^D1^ |
| Malta | -3.401** ^D1^ | -2.710* ^D1^ | -3.367** ^D1^ | -2.590* ^D1^ |
| Moldova | -2.782* | -3.744*** ^D1^ | -2.708* | -3.719*** ^D1^ |
| Netherland | -5.036*** ^D1^ | -2.664* | -5.135*** ^D1^ | -2.892** |
| North Macedonia | -3.042** | -3.975*** ^D1^ | -2.916** | -3.971*** ^D1^ |
| Norway | -3.625*** | -4.066*** ^D1^ | -3.727*** | -4.068*** ^D1^ |
| Poland | -3.404** | -4.498*** ^D1^ | -3.382** | -4.548*** ^D1^ |
| Portugal | -3.565*** | -3.080** ^D1^ | -3.568*** | -3.130** ^D1^ |
| Romania | -4.298*** ^D1^ | -3.189** ^D1^ | -4.311*** ^D1^ | -3.145** ^D1^ |
| Russia | -4.806*** ^D1^ | -3.023** | -4.955*** ^D1^ | -3.001** |
| Slovak republic | -2.652* | -3.988*** ^D1^ | -2.708* | -3.979*** ^D1^ |
| Slovenia | -3.277** | -3.726*** ^D1^ | -3.279** | -3.742*** ^D1^ |
| Spain | -4.207*** | -2.936** ^D1^ | -4.218*** | -2.914** ^D1^ |
| Sweden | -4.424*** ^D1^ | -3.778*** ^D1^ | -4.490*** ^D1^ | -3.773*** ^D1^ |
| Switzerland | -5.963*** ^D1^ | -5.175*** ^D1^ | -5.855*** ^D1^ | -5.454*** ^D1^ |
| Turkey | -3.226** ^D1^ | -2.679* | -3.178** ^D1^ | -2.612* |
| UK | -2.831* | -4.037*** ^D1^ | -2.822* | -4.046*** ^D1^ |
| Ukraine | -4.204*** ^D1^ | -3.512*** ^D1^ | -4.196*** ^D1^ | -3.403** ^D1^ |

Notes: *, denotes significance at the 10% level, **, denotes significance at the 5% level,***, denotes significance at the 1% level. D1= first difference of variable; D2= second difference of variable; D3= third difference of variable

### Table 02: Unit root test results – Africa Countries

| **Country** | **Augmented Dickey–Fuller** | | **Phillips–Perron** | |
| --- | --- | --- | --- | --- |
|  | **FDI** | **NE** | **FDI** | **NE** |
| Angola | -2.781* | -3.772**^D1^ | -2.721* | -3.753*** ^D1^ |
| Botswana | -4.905*** ^D1^ | -4.197*** ^D1^ | -5.671*** ^D1^ | -4.197*** ^D1^ |
| Cabo Verde (Cape Verde) | -2.816* | -3.871*** ^D1^ | -2.801* | -3.795*** ^D1^ |
| Djibouti | -4.184*** ^D1^ | -2.819* | -4.215*** ^D1^ | -2.751* |
| Egypt, Arab Rep. | -3.219** ^D1^ | -3.556*** ^D1^ | -3.236** ^D1^ | -3.532*** ^D1^ |
| Eswatini (Swasiland) | -4.764*** | -3.813*** ^D1^ | -4.721*** | -3.810*** ^D1^ |
| Ethiopia | -2.777* ^D1^ | -5.282*** ^D2^ | -2.800* ^D1^ | -5.681*** ^D2^ |
| Ghana | -3.776*** ^D1^ | -4.149*** ^D1^ | -3.890*** ^D1^ | -4.156*** ^D1^ |
| Kenya | -3.642*** ^D1^ | -4.508*** ^D1^ | -3.588*** ^D1^ | -4.689*** ^D1^ |
| Madagascar | -4.634*** ^D2^ | -2.660* ^D1^ | -4.725*** ^D2^ | -2.592* ^D1^ |
| Malawi | -3.909*** | -5.587*** ^D1^ | -3.911*** | -6.154*** ^D1^ |
| Mauritius | -6.048*** ^D1^ | -4.787*** ^D1^ | -6.056*** ^D1^ | -4.826*** ^D1^ |
| Morocco | -3.270** | -4.000*** ^D1^ | -3.265** | -3.997*** ^D1^ |
| Namibia | -5.012*** ^D1^ | -3.772*** ^D2^ | -5.119*** ^D1^ | -3.813*** ^D2^ |
| Nigeria | -4.526*** ^D1^ | -3.422** ^D1^ | -4.513*** ^D1^ | -3.363** ^D1^ |
| Sao Tome and Principe | -3.385** | -4.518*** ^D1^ | -3.367** | -4.552*** ^D1^ |
| Seychelles | -3.951*** | -6.020*** ^D1^ | -3.946*** | -6.522*** ^D1^ |
| Sierra Leone | -4.186*** ^D1^ | -4.543*** ^D1^ | -4.204*** ^D1^ | -4.942*** ^D1^ |
| South Africa | -3.089** | -3.643*** ^D2^ | -3.089** | -3.562*** ^D2^ |
| Tunisia | -3.183** | -2.670* ^D1^ | -3.233** | -2.651* ^D1^ |
| Zambia | -6.202*** ^D1^ | -3.025** ^D1^ | -6.046*** ^D1^ | -2.851* ^D1^ |

Notes: *, denotes significance at the 10% level, **, denotes significance at the 5% level,***, denotes significance at the 1% level. D1= first difference of variable; D2= second difference of variable; D3= third difference of variable

### Table 03: Unit root test results - North America Countries

| **Country** | **Augmented Dickey–Fuller** | | **Phillips–Perron** | |
| --- | --- | --- | --- | --- |
|  | **FDI** | **NE** | **FDI** | **NE** |
| Antigua and Barbuda | -3.839*** ^D1^ | -3.484*** ^D1^ | -3.838*** ^D1^ | -3.454*** ^D1^ |
| Bahamas | -4.769*** ^D1^ | -3.397** | -4.716*** ^D1^ | -3.276** |
| Belize | -3.128** | -3.701*** ^D1^ | -3.076** | -3.647*** ^D1^ |
| Canada | -3.054** ^D1^ | -4.007*** ^D1^ | -2.995** ^D1^ | -4.015*** ^D1^ |
| Costa Rica | -3.112**^D1^ | -4.610*** ^D1^ | -3.016** ^D1^ | -4.724*** ^D1^ |
| Dominica | -2.848* | -4.077*** ^D1^ | -2.861* | -4.092*** ^D1^ |
| Dominican Republic | -6.150*** ^D1^ | -4.737*** ^D1^ | -6.767*** ^D1^ | -4.717*** ^D1^ |
| Grenada | -3.567*** ^D1^ | -3.956*** ^D1^ | -3.575*** ^D1^ | -3.962*** ^D1^ |
| Guatemala | -4.833*** | -3.329** ^D1^ | -4.895*** | -3.210** ^D1^ |
| Jamaica | -4.186*** ^D1^ | -4.157*** ^D1^ | -4.186*** ^D1^ | -4.231*** ^D1^ |
| Mexico | -3.807*** | -5.164***^D2^ | -3.803*** | -5.453*** ^D2^ |
| Nicaragua | -3.148** ^D1^ | -3.289** ^D1^ | -3.133** ^D1^ | -3.274** ^D1^ |
| Panama | -3.909*** ^D2^ | -4.532*** ^D1^ | -3.770*** ^D2^ | -4.882*** ^D1^ |
| St. Lucia | -3.757*** ^D1^ | -3.211** ^D1^ | -3.750*** ^D1^ | -2.925**^D1^ |
| St. Vincent and the Grenadines | -3.011** | -2.893** ^D1^ | -3.029** | -2.807* ^D1^ |
| Trinidad and Tobago | -4.902*** ^D1^ | -5.061*** ^D1^ | -5.090*** ^D1^ | -5.096*** ^D1^ |
| United States | -2.662* | -4.035*** ^D1^ | -2.629* | -4.033*** ^D1^ |

Notes: *, denotes significance at the 10% level, **, denotes significance at the 5% level,***, denotes significance at the 1% level. D1= first difference of variable; D2= second difference of variable; D3= third difference of variable

### Table 04: Unit root test results - South America Countries

| **Country** | **Augmented Dickey–Fuller** | | **Phillips–Perron** | |
| --- | --- | --- | --- | --- |
|  | **FDI** | **NE** | **FDI** | **NE** |
| Argentina | -3.256** | -4.132***^D1^ | -3.214** | -4.134*** ^D1^ |
| Bolivia | -3.626***^D1^ | -3.520*** ^D1^ | -3.65***3 ^D1^ | -3.548*** ^D1^ |
| Brazil | -3.989*** ^D1^ | -3.176** ^D2^ | -3.962*** ^D1^ | -3.051** ^D2^ |
| Chile | -3.299** ^D1^ | -3.987*** ^D1^ | -3.225** ^D1^ | -3.930*** ^D1^ |
| Colombia | -4.989*** ^D1^ | -3.102** ^D1^ | -5.044*** ^D1^ | -3.030** ^D1^ |
| Ecuador | -2.874** | -4.492*** ^D1^ | -2.850* | -4.509*** ^D1^ |
| Guyana | -3.285** ^D3^ | -3.723*** ^D1^ | -3.256** ^D3^ | -3.293** ^D1^ |
| Paraguay | -8.883*** ^D1^ | -4.018*** | -8.234*** ^D1^ | -4.040*** |
| Peru | -4.344*** ^D1^ | -3.372** ^D1^ | -4.357*** ^D1^ | -3.349** ^D1^ |
| Uruguay | -3.729*** | -6.092*** ^D1^ | -3.734*** | -5.997*** ^D1^ |

Notes: *, denotes significance at the 10% level, **, denotes significance at the 5% level,***, denotes significance at the 1% level. D1= first difference of variable; D2= second difference of variable; D3= third difference of variable

### Table 05: Unit root test results - Asia and Oceania Countries

| **Country** | **Augmented Dickey–Fuller** | | **Phillips–Perron** | |
| --- | --- | --- | --- | --- |
|  | **FDI** | **NE** | **FDI** | **NE** |
| Armenia | -3.776***^D1^ | -3.002** ^D1^ | -3.786***^D1^ | -2.957**^D1^ |
| Australia | -3.032** | -4.083*** ^D1^ | -2.993** | -4.093*** ^D1^ |
| Azerbaijan | -4.958*** ^D1^ | -2.996** ^D1^ | -5.196*** ^D1^ | -2.981** ^D1^ |
| Bangladesh | -3.912*** ^D1^ | -3.695*** ^D1^ | -3.889*** ^D1^ | -3.681*** ^D1^ |
| Cambodia | -3.852*** ^D1^ | -3.681*** ^D1^ | -3.846*** ^D1^ | -3.132** ^D1^ |
| China | -4.701*** ^D1^ | -2.924** ^D1^ | -4.754*** ^D1^ | -2.869** ^D1^ |
| India | -4.016*** ^D1^ | -4.666***^D2^ | -4.007*** ^D1^ | -4.789*** ^D2^ |
| Indonesia | -5.484*** ^D1^ | -3.573*** ^D1^ | -5.672*** ^D1^ | -3.432*** ^D1^ |
| Israel | -6.279*** ^D1^ | -3.296** ^D1^ | -6.310*** ^D1^ | -3.140** ^D1^ |
| Japan | -5.304*** ^D1^ | -3.305** ^D1^ | -5.436*** ^D1^ | -3.261** ^D1^ |
| Kazakhstan | -2.665* | -4.893*** ^D1^ | -2.603* | -5.037*** ^D1^ |
| Korea Republic | -3.571*** | -4.207*** ^D1^ | -3.584*** | -4.210*** ^D1^ |
| Kuwait | -3.155** ^D1^ | -2.966** ^D1^ | -3.162** ^D1^ | -2.950** ^D1^ |
| Kyrgyz Republic | -2.818* | -3.847*** ^D1^ | -2.761* | -3.838*** ^D1^ |
| Malaysia | -2.781* | -4.102*** ^D1^ | -2.671* | -4.104*** ^D1^ |
| Maldives | -6.558*** ^D1^ | -4.190*** ^D1^ | -7.123*** ^D1^ | -4.194*** ^D1^ |
| Mongolia | -4.743*** ^D1^ | -2.719* ^D1^ | -4.766*** ^D1^ | -2.695* ^D1^ |
| Nepal | -7.108*** ^D1^ | -2.757* ^D1^ | -7.416*** ^D1^ | -2.705* ^D1^ |
| Oman | -4.815*** ^D1^ | -3.838*** ^D1^ | -4.851*** ^D1^ | -3.824*** ^D1^ |
| Pakistan | -2.800* ^D1^ | -3.477*** ^D1^ | -2.826* ^D1^ | -3.438*** ^D1^ |
| Philippines | -2.962* ^D1^ | -4.767***^D2^ | -3.012** ^D1^ | -4.838***^D2^ |
| Saudi Arabia | -5.030***^D2^ | -3.395**^D1^ | -5.108***^D2^ | -3.339**^D1^ |
| Singapore | -6.912*** ^D1^ | -4.321*** ^D1^ | -8.550*** ^D1^ | -4.551*** ^D1^ |
| Solomon Islands | -4.756*** ^D1^ | -3.610*** | -4.987*** ^D1^ | -3.569*** |
| Sri Lanka | -3.210**^D1^ | -4.399*** ^D1^ | -3.124** ^D1^ | -4.573*** ^D1^ |
| Thailand | -3.534*** | -3.533*** ^D1^ | -3.368** | -3.555*** ^D1^ |
| Vanuatu | -5.129*** ^D1^ | -3.048** | -5.309*** ^D1^ | -2.916** |
| Vietnam | -3.561*** ^D1^ | -3.648*** ^D1^ | -3.520*** ^D1^ | -3.670*** ^D1^ |

Notes: *, denotes significance at the 10% level, **, denotes significance at the 5% level,***, denotes significance at the 1% level. D1= first difference of variable; D2= second difference of variable; D3= third difference of variable

### Table 06: Levin-Lin-Chu unit root test results – All Region

|  | **All countries** | **Africa** | **Asia & Oceania** | **Europe** | **North America** | **South America** |
| --- | --- | --- | --- | --- | --- | --- |
| FDI |  |  |  |  | -4.2115*** |  |
| DFDI |  |  | -10.4928*** | -12.2168*** |  | -6.1636*** |
| DNE | -17.0406*** | -8.2751*** | -8.5542*** |  | -6.1153*** |  |
| DDFDI | -20.1965*** | -15.5094*** |  |  |  |  |
| DDNE |  |  |  | -17.9255*** |  | -6.8900*** |

Notes: ***, denote significance at 1%. DFDI = first difference of FDI; DDFDI = second difference of FDI; DNE = first difference of NE; DDNE = second difference of NE
