# Supplementary material for: From short to long term: Dynamic analysis of FDI and net export in global regions
Source: PLoS One. 2023 Sep 14;18(9):e0291301. doi: 10.1371/journal.pone.0291301 (PMC10501631; doi:10.1371/journal.pone.0291301)
Supplement: S4 Appendix — (DOCX) [file pone.0291301.s004.docx]

# Appendix S4. Lag length criteria results

## Table 01: Lag length criteria results for European countries.

|  | **lag** | **LL** | **LR** | **df** | **p** | **FPE** | **AIC** | **HQIC** | **SBIC** |
| --- | --- | --- | --- | --- | --- | --- | --- | --- | --- |
| Albania | 0 | -105.659 |  |  |  | 1.70E+09 | 26.9147 | 26.7808 | 26.9346 |
|  | 1 | -99.3027 | 12.712 | 4 | 0.013 | 1.00E+09 | 26.3257 | 25.9238 | 26.3853 |
|  | 2 | -97.2456 | 4.1142 | 4 | 0.391 | 2.30E+09 | 26.8114 | 26.1417 | 26.9107 |
|  | 3 | 59.8799 | 314.25 | 4 | 0 | 2.4e-07* | -11.47 | -12.4076 | -11.3309 |
|  | 4 | 435.834 | 751.91 | 4 | 0 | . | -104.959 | -106.03 | -104.8 |
|  | 5 | 444.184 | 16.699 | 4 | 0.002 | . | -107.046 | -108.118 | -106.887 |
|  | 6 | 449.657 | 10.947 | 4 | 0.027 | . | -108.414 | -109.486 | -108.255 |
|  | 7 | 455.518 | 11.722 | 4 | 0.02 | . | -109.879 | -110.951 | -109.721 |
|  | 8 | 463.585 | 16.134 | 4 | 0.003 | . | -111.896 | -112.968 | -111.737 |
|  | 9 | 460.156 | -6.8589 | 4 | . | . | -111.039 | -112.11 | -110.88 |
|  | 10 | 475.572 | 30.832* | 4 | 0 | . | -114.893* | -115.965* | -114.734* |
| Belarus | 0 | -129.324 |  |  |  | 6.20E+11 | 32.8309 | 32.697 | 32.8508 |
|  | 1 | -123.332 | 11.983 | 4 | 0.017 | 4.10E+11 | 32.3331 | 31.9312 | 32.3927 |
|  | 2 | -112.287 | 22.091 | 4 | 0 | 1.00E+11 | 30.5717 | 29.902 | 30.671 |
|  | 3 | . | . | 4 | . | -.000037* | . | . | . |
|  | 4 | 412.18 | . | 4 | . | . | -99.0449 | -100.117 | -98.886 |
|  | 5 | 410.263 | -3.8327 | 4 | . | . | -98.5658 | -99.6374 | -98.4069 |
|  | 6 | 415.183 | 9.84 | 4 | 0.043 | . | -99.7958 | -100.867 | -99.6369 |
|  | 7 | 429.616 | 28.866 | 4 | 0 | . | -103.404 | -104.476 | -103.245 |
|  | 8 | 422.959 | -13.314 | 4 | . | . | -101.74 | -102.811 | -101.581 |
|  | 9 | 434.303 | 22.688* | 4 | 0 | . | -104.576 | -105.647 | -104.417 |
|  | 10 | 434.303 | 0 | 4 | . | . | -104.576* | -105.647* | -104.417* |
| Bulgaria | 0 | -129.693 |  |  |  | 6.80E+11 | 32.9233 | 32.7894 | 32.9432 |
|  | 1 | -125.242 | 8.9025 | 4 | 0.064 | 6.60E+11 | 32.8105 | 32.4087 | 32.8701 |
|  | 2 | -124.886 | 0.71136 | 4 | 0.95 | 2.30E+12 | 33.7216 | 33.0518 | 33.8209 |
|  | 3 | . | . | 4 | . | 0* | . | . | . |
|  | 4 | 417.89 | . | 4 | . | . | -100.473 | -101.544 | -100.314 |
|  | 5 | 424.511 | 13.242 | 4 | 0.01 | . | -102.128 | -103.199 | -101.969 |
|  | 6 | 422.034 | -4.9536 | 4 | . | . | -101.509 | -102.58 | -101.35 |
|  | 7 | 427.274 | 10.479* | 4 | 0.033 | . | -102.818* | -103.89* | -102.66* |
|  | 8 | 427.104 | -0.33956 | 4 | . | . | -102.776 | -103.848 | -102.617 |
|  | 9 | 417.054 | -20.1 | 4 | . | . | -100.264 | -101.335 | -100.105 |
|  | 10 | 420.474 | 6.8393 | 4 | 0.145 | . | -101.119 | -102.19 | -100.96 |
| Croatia | 0 | -139.849 |  |  |  | 8.70E+12 | 35.4622 | 35.3282 | 35.482 |
|  | 1 | -132.63 | 14.438 | 4 | 0.006 | 4.20E+12 | 34.6574 | 34.2556 | 34.717 |
|  | 2 | -127.498 | 10.264 | 4 | 0.036 | 4.50E+12 | 34.3744 | 33.7047 | 34.4737 |
|  | 3 | . | . | 4 | . | 0* | . | . | . |
|  | 4 | 391.776 | . | 4 | . | . | -93.944 | -95.0156 | -93.7851 |
|  | 5 | 431.989 | 80.426 | 4 | 0 | . | -103.997 | -105.069 | -103.838 |
|  | 6 | 431.604 | -0.77135 | 4 | . | . | -103.901 | -104.972 | -103.742 |
|  | 7 | 425.583 | -12.041 | 4 | . | . | -102.396 | -103.467 | -102.237 |
|  | 8 | 425.504 | -0.15895 | 4 | . | . | -102.376 | -103.448 | -102.217 |
|  | 9 | 422.306 | -6.3953 | 4 | . | . | -101.577 | -102.648 | -101.418 |
|  | 10 | 434.893 | 25.174* | 4 | 0 | . | -104.723* | -105.795* | -104.564* |
| Cyprus | 0 | -150.559 |  |  |  | 1.30E+14 | 38.1396 | 38.0057 | 38.1595 |
|  | 1 | -144.748 | 11.62 | 4 | 0.02 | 8.60E+13 | 37.6871 | 37.2852 | 37.7467 |
|  | 2 | -142.133 | 5.2303 | 4 | 0.264 | 1.70E+14 | 38.0333 | 37.3635 | 38.1326 |
|  | 3 | . | . | 4 | . | 0* | . | . | . |
|  | 4 | 395.241 | . | 4 | . | . | -94.8104 | -95.882 | -94.6515 |
|  | 5 | 385.132 | -20.22 | 4 | . | . | -92.2829 | -93.3545 | -92.124 |
|  | 6 | 381.432 | -7.3999 | 4 | . | . | -91.3579 | -92.4295 | -91.199 |
|  | 7 | 386.213 | 9.5625 | 4 | 0.048 | . | -92.5532 | -93.6248 | -92.3944 |
|  | 8 | 398.35 | 24.274 | 4 | 0 | . | -95.5875 | -96.6591 | -95.4287 |
|  | 9 | 405.275 | 13.849* | 4 | 0.008 | . | -97.3187 | -98.3903 | -97.1598 |
|  | 10 | 406.168 | 1.7866 | 4 | 0.775 | . | -97.542* | -98.6136* | -97.3831* |
| Czech Republic | 0 | -144.686 |  |  |  | 2.90E+13 | 36.6716 | 36.5376 | 36.6914 |
|  | 1 | -143.218 | 2.9376 | 4 | 0.568 | 5.90E+13 | 37.3044 | 36.9025 | 37.364 |
|  | 2 | -141.053 | 4.3284 | 4 | 0.363 | 1.30E+14 | 37.7633 | 37.0936 | 37.8626 |
|  | 3 | . | . | 4 | . | -.021187* | . | . | . |
|  | 4 | 403.848 | . | 4 | . | . | -96.9621 | -98.0337 | -96.8032 |
|  | 5 | 400.6 | -6.4963 | 4 | . | . | -96.1501 | -97.2217 | -95.9912 |
|  | 6 | 411.48 | 21.759 | 4 | 0 | . | -98.8699 | -99.9415 | -98.7111 |
|  | 7 | 405.464 | -12.031 | 4 | . | . | -97.3661 | -98.4377 | -97.2072 |
|  | 8 | 416.419 | 21.908* | 4 | 0 | . | -100.105 | -101.176 | -99.9458 |
|  | 9 | 416.033 | -0.77138 | 4 | . | . | -100.008 | -101.08 | -99.8493 |
|  | 10 | 416.456 | 0.84526 | 4 | 0.932 | . | -100.114* | -101.185* | -99.955* |
| Denmark | 0 | -151.997 |  |  |  | 1.80E+14 | 38.4993 | 38.3654 | 38.5192 |
|  | 1 | -150.353 | 3.2878 | 4 | 0.511 | 3.50E+14 | 39.0884 | 38.6865 | 39.1479 |
|  | 2 | -145.618 | 9.4719 | 4 | 0.05 | 4.20E+14 | 38.9044 | 38.2346 | 39.0037 |
|  | 3 | . | . | 4 | . | -.340335* | . | . | . |
|  | 4 | 401.085 | . | 4 | . | . | -96.2713 | -97.3429 | -96.1124 |
|  | 5 | 396.975 | -8.22 | 4 | . | . | -95.2438 | -96.3154 | -95.0849 |
|  | 6 | 401.727 | 9.5039 | 4 | 0.05 | . | -96.4318 | -97.5034 | -96.2729 |
|  | 7 | 408.318 | 13.182 | 4 | 0.01 | . | -98.0795 | -99.1511 | -97.9207 |
|  | 8 | 405.993 | -4.6498 | 4 | . | . | -97.4983 | -98.5699 | -97.3394 |
|  | 9 | 411.176 | 10.366* | 4 | 0.035 | . | -98.7941 | -99.8657 | -98.6352 |
|  | 10 | 411.176 | 0 | 4 | . | . | -98.7941* | -99.8657* | -98.6352* |
| Estonia | 0 | -128.931 |  |  |  | 5.60E+11 | 32.7328 | 32.5988 | 32.7526 |
|  | 1 | -125.382 | 7.0977 | 4 | 0.131 | 6.80E+11 | 32.8456 | 32.4437 | 32.9052 |
|  | 2 | -124.402 | 1.9612 | 4 | 0.743 | 2.10E+12 | 33.6004 | 32.9307 | 33.6997 |
|  | 3 | 30.3821 | 309.57 | 4 | 0 | .000388* | -4.09553 | -5.03318 | -3.95651 |
|  | 4 | 435.812 | 810.86 | 4 | 0 | . | -104.953 | -106.025 | -104.794 |
|  | 5 | 434.3 | -3.0247 | 4 | . | . | -104.575 | -105.646 | -104.416 |
|  | 6 | 444.316 | 20.033* | 4 | 0 | . | -107.079 | -108.151 | -106.92 |
|  | 7 | 445.458 | 2.2838 | 4 | 0.684 | . | -107.365 | -108.436 | -107.206 |
|  | 8 | 445.458 | 0 | 4 | . | . | -107.365 | -108.436 | -107.206 |
|  | 9 | 445.458 | 0 | 4 | . | . | -107.365 | -108.436 | -107.206 |
|  | 10 | 445.458 | 0 | 4 | . | . | -107.365* | -108.436* | -107.206* |
| Finland | 0 | -156.604 |  |  |  | 5.70E+14 | 39.651 | 39.517 | 39.6708 |
|  | 1 | -152.162 | 8.8831 | 4 | 0.064 | 5.50E+14 | 39.5406 | 39.1387 | 39.6002 |
|  | 2 | -147.942 | 8.4402 | 4 | 0.077 | 7.40E+14 | 39.4855 | 38.8158 | 39.5848 |
|  | 3 | 1.76421 | 299.41 | 4 | 0 | .496235* | 3.05895 | 2.1213 | 3.19797 |
|  | 4 | 403.123 | 802.72 | 4 | 0 | . | -96.7807 | -97.8523 | -96.6219 |
|  | 5 | 406.5 | 6.7535 | 4 | 0.15 | . | -97.6249 | -98.6965 | -97.466 |
|  | 6 | 413.119 | 13.238 | 4 | 0.01 | . | -99.2797* | -100.351* | -99.1208* |
|  | 7 | 372.458 | -81.321 | 4 | . | . | -89.1145 | -90.1861 | -88.9557 |
|  | 8 | 409.107 | 73.298* | 4 | 0 | . | -98.2768 | -99.3484 | -98.1179 |
|  | 9 | 398.03 | -22.155 | 4 | . | . | -95.5075 | -96.5791 | -95.3486 |
|  | 10 | 396.544 | -2.9718 | 4 | . | . | -95.136 | -96.2076 | -94.9771 |
| France | 0 | -179.571 |  |  |  | 1.80E+17 | 45.3927 | 45.2587 | 45.4125 |
|  | 1 | -178.603 | 1.9351 | 4 | 0.748 | 4.10E+17 | 46.1508 | 45.7489 | 46.2103 |
|  | 2 | -175.534 | 6.1377 | 4 | 0.189 | 7.40E+17 | 46.3836 | 45.7138 | 46.4829 |
|  | 3 | . | . | 4 | . | -239.601* | . | . | . |
|  | 4 | 372.922 | . | 4 | . | . | -89.2305 | -90.3021 | -89.0716 |
|  | 5 | 390.085 | 34.325 | 4 | 0 | . | -93.5211 | -94.5927 | -93.3622 |
|  | 6 | 390.085 | 0 | 4 | . | . | -93.5211 | -94.5927 | -93.3622 |
|  | 7 | 390.085 | 0 | 4 | . | . | -93.5211* | -94.5927* | -93.3622* |
|  | 8 | 376.674 | -26.821 | 4 | . | . | -90.1685 | -91.2401 | -90.0096 |
|  | 9 | 374.03 | -5.2873 | 4 | . | . | -89.5076 | -90.5792 | -89.3487 |
|  | 10 | 383.728 | 19.396* | 4 | 0.001 | . | -91.9321 | -93.0037 | -91.7732 |
| Germany | 0 | -183.734 |  |  |  | 5.00E+17 | 46.4336 | 46.2997 | 46.4535 |
|  | 1 | -181.581 | 4.3064 | 4 | 0.366 | 8.60E+17 | 46.8953 | 46.4935 | 46.9549 |
|  | 2 | -176.126 | 10.91 | 4 | 0.028 | 8.50E+17 | 46.5316 | 45.8618 | 46.6309 |
|  | 3 | -20.8123 | 310.63 | 4 | 0 | 140.248* | 8.70306 | 7.76541 | 8.84209 |
|  | 4 | 350.083 | 741.79 | 4 | 0 | . | -83.5206 | -84.5922 | -83.3618 |
|  | 5 | 367.941 | 35.717 | 4 | 0 | . | -87.9853 | -89.0569 | -87.8264 |
|  | 6 | 365.118 | -5.6462 | 4 | . | . | -87.2795 | -88.3511 | -87.1206 |
|  | 7 | 366.81 | 3.3835 | 4 | 0.496 | . | -87.7024 | -88.774 | -87.5435 |
|  | 8 | 367.561 | 1.5025 | 4 | 0.826 | . | -87.8902 | -88.9618 | -87.7313 |
|  | 9 | 377.559 | 19.996* | 4 | 0.001 | . | -90.3897 | -91.4613 | -90.2308 |
|  | 10 | 377.559 | 0 | 4 | . | . | -90.3897* | -91.4613* | -90.2308* |
| Greece | 0 | -144.525 |  |  |  | 2.80E+13 | 36.6313 | 36.4974 | 36.6512 |
|  | 1 | -140.367 | 8.3174 | 4 | 0.081 | 2.90E+13 | 36.5917 | 36.1898 | 36.6512 |
|  | 2 | -139.914 | 0.90449 | 4 | 0.924 | 1.00E+14 | 37.4786 | 36.8089 | 37.5779 |
|  | 3 | . | . | 4 | . | 0* | . | . | . |
|  | 4 | 407.602 | . | 4 | . | . | -97.9006 | -98.9722 | -97.7417 |
|  | 5 | 400.788 | -13.629 | 4 | . | . | -96.1969 | -97.2685 | -96.038 |
|  | 6 | 409.279 | 16.984* | 4 | 0.002 | . | -98.3199 | -99.3915 | -98.161 |
|  | 7 | 407.951 | -2.6564 | 4 | . | . | -97.9878 | -99.0594 | -97.8289 |
|  | 8 | 407.972 | 0.04141 | 4 | 1 | . | -97.993 | -99.0646 | -97.8341 |
|  | 9 | 410.572 | 5.1994 | 4 | 0.267 | . | -98.6429 | -99.7145 | -98.484 |
|  | 10 | 410.961 | 0.77899 | 4 | 0.941 | . | -98.7403* | -99.8119* | -98.5814* |
| Hungary | 0 | -171.249 |  |  |  | 2.20E+16 | 43.3123 | 43.1783 | 43.3321 |
|  | 1 | -167.571 | 7.3568 | 4 | 0.118 | 2.60E+16 | 43.3927 | 42.9908 | 43.4523 |
|  | 2 | -158.261 | 18.62 | 4 | 0.001 | 9.80E+15 | 42.0652 | 41.3955 | 42.1645 |
|  | 3 | . | . | 4 | . | 0* | . | . | . |
|  | 4 | 373.929 | . | 4 | . | . | -89.4823 | -90.5539 | -89.3234 |
|  | 5 | 374.852 | 1.8453 | 4 | 0.764 | . | -89.7129 | -90.7845 | -89.554 |
|  | 6 | 382.49 | 15.277 | 4 | 0.004 | . | -91.6225 | -92.6941 | -91.4637 |
|  | 7 | 384.276 | 3.5723 | 4 | 0.467 | . | -92.0691 | -93.1407 | -91.9102 |
|  | 8 | 387.352 | 6.1515 | 4 | 0.188 | . | -92.838 | -93.9096 | -92.6791 |
|  | 9 | 396.57 | 18.436* | 4 | 0.001 | . | -95.1425* | -96.2141* | -94.9836* |
|  | 10 | 394.401 | -4.3368 | 4 | . | . | -94.6004 | -95.672 | -94.4415 |
| Iceland | 0 | -121.52 |  |  |  | 7.3e+12* | 35.2914 | 35.1004 | 35.276 |
|  | 1 | -118.141 | 6.7583 | 4 | 0.149 | 9.80E+12 | 35.4688 | 34.8958 | 35.4224 |
|  | 2 | -112.087 | 12.107 | 4 | 0.017 | 1.00E+13 | 34.8821 | 33.927 | 34.8048 |
|  | 3 | 356.98 | 938.13 | 4 | 0 | . | -97.9943 | -99.3314 | -98.1025 |
|  | 4 | 379.273 | 44.585 | 4 | 0 | . | -104.364 | -105.701 | -104.472 |
|  | 5 | 376.657 | -5.2323 | 4 | . | . | -103.616 | -104.953 | -103.724 |
|  | 6 | 370.44 | -12.433 | 4 | . | . | -101.84 | -103.177 | -101.948 |
|  | 7 | 374.416 | 7.9506 | 4 | 0.093 | . | -102.976 | -104.313 | -103.084 |
|  | 8 | 367.706 | -13.42 | 4 | . | . | -101.059 | -102.396 | -101.167 |
|  | 9 | 378.862 | 22.314 | 4 | 0 | . | -104.246 | -105.584 | -104.355 |
|  | 10 | 385.104 | 12.483* | 4 | 0.014 | . | -106.03* | -107.367* | -106.138* |
| Italy | 0 | -175.911 |  |  |  | 7.10E+16 | 44.4777 | 44.3437 | 44.4975 |
|  | 1 | -170.766 | 10.289 | 4 | 0.036 | 5.80E+16 | 44.1915 | 43.7896 | 44.2511 |
|  | 2 | -168.464 | 4.6049 | 4 | 0.33 | 1.30E+17 | 44.6159 | 43.9461 | 44.7152 |
|  | 3 | . | . | 4 | . | -92.0783* | . | . | . |
|  | 4 | 363.255 | . | 4 | . | . | -86.8138 | -87.8854 | -86.655 |
|  | 5 | 375.263 | 24.014 | 4 | 0 | . | -89.8156 | -90.8872 | -89.6568 |
|  | 6 | 384.55 | 18.575 | 4 | 0.001 | . | -92.1375 | -93.2091 | -91.9786 |
|  | 7 | 383.281 | -2.5375 | 4 | . | . | -91.8203 | -92.8919 | -91.6614 |
|  | 8 | 396.628 | 26.694* | 4 | 0 | . | -95.1571* | -96.2287* | -94.9982* |
|  | 9 | 395.602 | -2.0522 | 4 | . | . | -94.9005 | -95.9721 | -94.7416 |
|  | 10 | 393.456 | -4.2923 | 4 | . | . | -94.364 | -95.4356 | -94.2051 |
| Latvia | 0 | -113.506 |  |  |  | 1.20E+10 | 28.8766 | 28.7426 | 28.8964 |
|  | 1 | -110.419 | 6.1747 | 4 | 0.186 | 1.60E+10 | 29.1047 | 28.7029 | 29.1643 |
|  | 2 | -108.008 | 4.8217 | 4 | 0.306 | 3.40E+10 | 29.502 | 28.8322 | 29.6013 |
|  | 3 | 59.8596 | 335.74 | 4 | 0 | 2.4e-07* | -11.4649 | -12.4026 | -11.3259 |
|  | 4 | 409.161 | 698.6 | 4 | 0 | . | -98.2902 | -99.3618 | -98.1313 |
|  | 5 | 448.611 | 78.9* | 4 | 0 | . | -108.153* | -109.224* | -107.994* |
|  | 6 | 439.87 | -17.481 | 4 | . | . | -105.968 | -107.039 | -105.809 |
|  | 7 | 440.736 | 1.7303 | 4 | 0.785 | . | -106.184 | -107.256 | -106.025 |
|  | 8 | 438.01 | -5.4513 | 4 | . | . | -105.502 | -106.574 | -105.344 |
|  | 9 | 441.918 | 7.8161 | 4 | 0.099 | . | -106.48 | -107.551 | -106.321 |
|  | 10 | 430.791 | -22.254 | 4 | . | . | -103.698 | -104.769 | -103.539 |
| Lithuania | 0 | -130.194 |  |  |  | 7.70E+11 | 33.0485 | 32.9146 | 33.0684 |
|  | 1 | -128.012 | 4.3634 | 4 | 0.359 | 1.30E+12 | 33.5031 | 33.1012 | 33.5627 |
|  | 2 | -124.758 | 6.508 | 4 | 0.164 | 2.30E+12 | 33.6896 | 33.0198 | 33.7889 |
|  | 3 | . | . | 4 | . | 0* | . | . | . |
|  | 4 | 427.639 | . | 4 | . | . | -102.91 | -103.981 | -102.751 |
|  | 5 | 433.432 | 11.585 | 4 | 0.021 | . | -104.358 | -105.43 | -104.199 |
|  | 6 | 439.499 | 12.135 | 4 | 0.016 | . | -105.875 | -106.946 | -105.716 |
|  | 7 | 439.463 | -0.07286 | 4 | . | . | -105.866 | -106.937 | -105.707 |
|  | 8 | 436.191 | -6.543 | 4 | . | . | -105.048 | -106.119 | -104.889 |
|  | 9 | 432.065 | -8.2536 | 4 | . | . | -104.016 | -105.088 | -103.857 |
|  | 10 | 459.412 | 54.695* | 4 | 0 | . | -110.853* | -111.925* | -110.694* |
| Malta | 0 | -133.091 |  |  |  | 1.60E+12 | 33.7727 | 33.6387 | 33.7926 |
|  | 1 | -131.152 | 3.8782 | 4 | 0.423 | 2.90E+12 | 34.2879 | 33.8861 | 34.3475 |
|  | 2 | -129.801 | 2.7019 | 4 | 0.609 | 8.00E+12 | 34.9502 | 34.2804 | 35.0495 |
|  | 3 | . | . | 4 | . | -.000374* | . | . | . |
|  | 4 | 427.607 | . | 4 | . | . | -102.902 | -103.973 | -102.743 |
|  | 5 | 444.036 | 32.857 | 4 | 0 | . | -107.009* | -108.08* | -106.85* |
|  | 6 | 413.803 | -60.464 | 4 | . | . | -99.4508 | -100.522 | -99.2919 |
|  | 7 | 413.898 | 0.19034 | 4 | 0.996 | . | -99.4746 | -100.546 | -99.3157 |
|  | 8 | 422.186 | 16.575 | 4 | 0.002 | . | -101.546 | -102.618 | -101.388 |
|  | 9 | 414.919 | -14.534 | 4 | . | . | -99.7297 | -100.801 | -99.5708 |
|  | 10 | 426.703 | 23.568* | 4 | 0 | . | -102.676 | -103.747 | -102.517 |
| Moldova | 0 | -109.896 |  |  |  | 4.80E+09 | 27.9739 | 27.84 | 27.9938 |
|  | 1 | -101.503 | 16.786 | 4 | 0.002 | 1.70E+09 | 26.8757 | 26.4739 | 26.9353 |
|  | 2 | -99.0301 | 4.9457 | 4 | 0.293 | 3.60E+09 | 27.2575 | 26.5878 | 27.3568 |
|  | 3 | . | . | 4 | . | 0* | . | . | . |
|  | 4 | 444.589 | . | 4 | . | . | -107.147 | -108.219 | -106.988 |
|  | 5 | 451.931 | 14.683 | 4 | 0.005 | . | -108.983 | -110.054 | -108.824 |
|  | 6 | 426.655 | -50.552 | 4 | . | . | -102.664 | -103.735 | -102.505 |
|  | 7 | 437.546 | 21.783 | 4 | 0 | . | -105.387 | -106.458 | -105.228 |
|  | 8 | 449.048 | 23.003 | 4 | 0 | . | -108.262 | -109.334 | -108.103 |
|  | 9 | 454.475 | 10.854* | 4 | 0.028 | . | -109.619 | -110.69 | -109.46 |
|  | 10 | 457.584 | 6.2178 | 4 | 0.183 | . | -110.396* | -111.468* | -110.237* |
| Netherland | 0 | -195.565 |  |  |  | 9.70E+18 | 49.3912 | 49.2573 | 49.4111 |
|  | 1 | -194.435 | 2.2591 | 4 | 0.688 | 2.10E+19 | 50.1088 | 49.707 | 50.1684 |
|  | 2 | -191.182 | 6.5069 | 4 | 0.164 | 3.70E+19 | 50.2955 | 49.6257 | 50.3948 |
|  | 3 | . | . | 4 | . | -7206.44* | . | . | . |
|  | 4 | 358.991 | . | 4 | . | . | -85.7478 | -86.8194 | -85.5889 |
|  | 5 | 365.459 | 12.936* | 4 | 0.012 | . | -87.3649* | -88.4365* | -87.206* |
|  | 6 | 359.227 | -12.465 | 4 | . | . | -85.8067 | -86.8783 | -85.6478 |
|  | 7 | 363.602 | 8.7512 | 4 | 0.068 | . | -86.9006 | -87.9722 | -86.7417 |
|  | 8 | 364.779 | 2.3524 | 4 | 0.671 | . | -87.1946 | -88.2662 | -87.0358 |
|  | 9 | . | . | 4 | . | . | . | . | . |
|  | 10 | 360.179 | . | 4 | . | . | -86.0447 | -87.1163 | -85.8858 |
| North Macedonia | 0 | -105.03 |  |  |  | 1.40E+09 | 26.7575 | 26.6236 | 26.7774 |
|  | 1 | -96.6862 | 16.688 | 4 | 0.002 | 5.20E+08 | 25.6715 | 25.2697 | 25.7311 |
|  | 2 | -95.2383 | 2.8958 | 4 | 0.575 | 1.40E+09 | 26.3096 | 25.6398 | 26.4089 |
|  | 3 | . | . | 4 | . | 0* | . | . | . |
|  | 4 | 454.113 | . | 4 | . | . | -109.528 | -110.6 | -109.369 |
|  | 5 | 454.944 | 1.6618 | 4 | 0.798 | . | -109.736 | -110.808 | -109.577 |
|  | 6 | 460.572 | 11.256 | 4 | 0.024 | . | -111.143* | -112.214* | -110.984* |
|  | 7 | 445.047 | -31.049 | 4 | . | . | -107.262 | -108.333 | -107.103 |
|  | 8 | 454.127 | 18.16* | 4 | 0.001 | . | -109.532 | -110.603 | -109.373 |
|  | 9 | 453.152 | -1.9495 | 4 | . | . | -109.288 | -110.36 | -109.129 |
|  | 10 | 457.564 | 8.8224 | 4 | 0.066 | . | -110.391 | -111.462 | -110.232 |
| Norway | 0 | -170.756 |  |  |  | 2.00E+16 | 43.1891 | 43.0551 | 43.2089 |
|  | 1 | -166.922 | 7.6679 | 4 | 0.105 | 2.20E+16 | 43.2306 | 42.8287 | 43.2902 |
|  | 2 | -163.916 | 6.0126 | 4 | 0.198 | 4.00E+16 | 43.479 | 42.8092 | 43.5783 |
|  | 3 | -0.75743 | 326.32 | 4 | 0 | .932116* | 3.68936 | 2.7517 | 3.82838 |
|  | 4 | 377.149 | 755.81 | 4 | 0 | . | -90.2873 | -91.3589 | -90.1284 |
|  | 5 | 382.907 | 11.516 | 4 | 0.021 | . | -91.7268 | -92.7984 | -91.5679 |
|  | 6 | 381.422 | -2.9701 | 4 | . | . | -91.3555 | -92.4271 | -91.1967 |
|  | 7 | 394.336 | 25.827 | 4 | 0 | . | -94.5839 | -95.6555 | -94.4251 |
|  | 8 | 394.501 | 0.33005 | 4 | 0.988 | . | -94.6252 | -95.6968 | -94.4663 |
|  | 9 | 393.509 | -1.9842 | 4 | . | . | -94.3772 | -95.4488 | -94.2183 |
|  | 10 | 401.619 | 16.221* | 4 | 0.003 | . | -96.4048* | -97.4764* | -96.2459* |
| Poland | 0 | -159.784 |  |  |  | 1.30E+15 | 40.446 | 40.312 | 40.4658 |
|  | 1 | -158.212 | 3.1428 | 4 | 0.534 | 2.50E+15 | 41.0531 | 40.6513 | 41.1127 |
|  | 2 | -147.892 | 20.641 | 4 | 0 | 7.30E+14 | 39.473 | 38.8033 | 39.5723 |
|  | 3 | . | . | 4 | . | -.011706* | . | . | . |
|  | 4 | 403.309 | . | 4 | . | . | -96.8272 | -97.8988 | -96.6683 |
|  | 5 | 396.693 | -13.231 | 4 | . | . | -95.1734 | -96.245 | -95.0145 |
|  | 6 | 404.81 | 16.233 | 4 | 0.003 | . | -97.2024 | -98.274 | -97.0436 |
|  | 7 | 405.145 | 0.66955 | 4 | 0.955 | . | -97.2861 | -98.3577 | -97.1273 |
|  | 8 | 411.458 | 12.628* | 4 | 0.013 | . | -98.8646 | -99.9362 | -98.7057 |
|  | 9 | 412.206 | 1.4963 | 4 | 0.827 | . | -99.0516* | -100.123* | -98.8927* |
|  | 10 | 402.957 | -18.5 | 4 | . | . | -96.7392 | -97.8108 | -96.5803 |
| Protugal | 0 | -151.985 |  |  |  | 1.80E+14 | 38.4964 | 38.3624 | 38.5162 |
|  | 1 | -147.28 | 9.4118 | 4 | 0.052 | 1.60E+14 | 38.3199 | 37.918 | 38.3795 |
|  | 2 | -145.977 | 2.6059 | 4 | 0.626 | 4.50E+14 | 38.9941 | 38.3244 | 39.0934 |
|  | 3 | . | . | 4 | . | 0* | . | . | . |
|  | 4 | 390.746 | . | 4 | . | . | -93.6864 | -94.758 | -93.5275 |
|  | 5 | 412.255 | 43.018 | 4 | 0 | . | -99.0637 | -100.135 | -98.9048 |
|  | 6 | 408.966 | -6.5775 | 4 | . | . | -98.2415 | -99.3131 | -98.0826 |
|  | 7 | 403.534 | -10.864 | 4 | . | . | -96.8835 | -97.9551 | -96.7246 |
|  | 8 | 407.327 | 7.5855 | 4 | 0.108 | . | -97.8317 | -98.9033 | -97.6728 |
|  | 9 | 415.069 | 15.484* | 4 | 0.004 | . | -99.7672 | -100.839 | -99.6083 |
|  | 10 | 415.069 | 0 | 4 | . | . | -99.7672* | -100.839* | -99.6083* |
| Romania | 0 | -145.899 |  |  |  | 3.90E+13 | 36.9749 | 36.8409 | 36.9947 |
|  | 1 | -144.424 | 2.9518 | 4 | 0.566 | 8.00E+13 | 37.6059 | 37.204 | 37.6655 |
|  | 2 | -136.72 | 15.407 | 4 | 0.004 | 4.50E+13 | 36.68 | 36.0103 | 36.7793 |
|  | 3 | . | . | 4 | . | 0* | . | . | . |
|  | 4 | 397.962 | . | 4 | . | . | -95.4904 | -96.562 | -95.3315 |
|  | 5 | 408.196 | 20.469 | 4 | 0 | . | -98.049 | -99.1206 | -97.8901 |
|  | 6 | 409.274 | 2.1552 | 4 | 0.707 | . | -98.3184 | -99.39 | -98.1595 |
|  | 7 | 416.547 | 14.547 | 4 | 0.006 | . | -100.137 | -101.208 | -99.9779 |
|  | 8 | 421.416 | 9.7372 | 4 | 0.045 | . | -101.354 | -102.426 | -101.195 |
|  | 9 | 416.626 | -9.5787 | 4 | . | . | -100.157 | -101.228 | -99.9977 |
|  | 10 | 429.545 | 25.838* | 4 | 0 | . | -103.386* | -104.458* | -103.227* |
| Russia | 0 | -185.896 |  |  |  | 8.60E+17 | 46.9741 | 46.8401 | 46.994 |
|  | 1 | -184.669 | 2.4547 | 4 | 0.653 | 1.90E+18 | 47.6673 | 47.2654 | 47.7268 |
|  | 2 | -177.017 | 15.304 | 4 | 0.004 | 1.10E+18 | 46.7542 | 46.0845 | 46.8535 |
|  | 3 | -17.2109 | 319.61 | 4 | 0 | 57.0014* | 7.80273 | 6.86508 | 7.94175 |
|  | 4 | 372.413 | 779.25 | 4 | 0 | . | -89.1034 | -90.175 | -88.9445 |
|  | 5 | 374.069 | 3.3107 | 4 | 0.507 | . | -89.5172 | -90.5888 | -89.3583 |
|  | 6 | 377.7 | 7.2634 | 4 | 0.123 | . | -90.4251* | -91.4967* | -90.2662* |
|  | 7 | 368.218 | -18.964 | 4 | . | . | -88.0546 | -89.1262 | -87.8957 |
|  | 8 | 369.691 | 2.9457 | 4 | 0.567 | . | -88.4228 | -89.4944 | -88.2639 |
|  | 9 | 376.079 | 12.775* | 4 | 0.012 | . | -90.0197 | -91.0913 | -89.8608 |
|  | 10 | 363.817 | -24.524 | 4 | . | . | -86.9542 | -88.0258 | -86.7953 |
| Slovak republic | 0 | -140.61 |  |  |  | 1.00E+13 | 35.6524 | 35.5185 | 35.6723 |
|  | 1 | -138.302 | 4.6148 | 4 | 0.329 | 1.70E+13 | 36.0756 | 35.6737 | 36.1352 |
|  | 2 | -136.34 | 3.9256 | 4 | 0.416 | 4.10E+13 | 36.5849 | 35.9151 | 36.6842 |
|  | 3 | . | . | 4 | . | 0* | . | . | . |
|  | 4 | 426.184 | . | 4 | . | . | -102.546* | -103.618* | -102.387* |
|  | 5 | 424.949 | -2.4712 | 4 | . | . | -102.237 | -103.309 | -102.078 |
|  | 6 | 423.965 | -1.9666 | 4 | . | . | -101.991 | -103.063 | -101.832 |
|  | 7 | 425.034 | 2.1377 | 4 | 0.71 | . | -102.259 | -103.33 | -102.1 |
|  | 8 | 420.03 | -10.01 | 4 | . | . | -101.007 | -102.079 | -100.849 |
|  | 9 | 420.03 | 0 | 4 | . | . | -101.007 | -102.079 | -100.849 |
|  | 10 | 420.03 | 0 | 4 | . | . | -101.007 | -102.079 | -100.849 |
| Slovenia | 0 | -118.667 |  |  |  | 4.30E+10 | 30.1667 | 30.0327 | 30.1865 |
|  | 1 | -111.393 | 14.546 | 4 | 0.006 | 2.10E+10 | 29.3484 | 28.9465 | 29.408 |
|  | 2 | -101.847 | 19.093 | 4 | 0.001 | 7.40E+09 | 27.9618 | 27.292 | 28.0611 |
|  | 3 | 43.5492 | 290.79 | 4 | 0 | .000014* | -7.3873 | -8.32495 | -7.24827 |
|  | 4 | 415.371 | 743.64 | 4 | 0 | . | -99.8427 | -100.914 | -99.6839 |
|  | 5 | 420.932 | 11.122 | 4 | 0.025 | . | -101.233 | -102.305 | -101.074 |
|  | 6 | 421.03 | 0.19561 | 4 | 0.996 | . | -101.257 | -102.329 | -101.099 |
|  | 7 | 436.627 | 31.194 | 4 | 0 | . | -105.157 | -106.228 | -104.998 |
|  | 8 | 430.613 | -12.027 | 4 | . | . | -103.653 | -104.725 | -103.494 |
|  | 9 | 441.63 | 22.033 | 4 | 0 | . | -106.407 | -107.479 | -106.249 |
|  | 10 | 450.545 | 17.829* | 4 | 0.001 | . | -108.636* | -109.708* | -108.477* |
| Spain | 0 | -173.429 |  |  |  | 3.80E+16 | 43.8571 | 43.7232 | 43.877 |
|  | 1 | -171.621 | 3.6159 | 4 | 0.46 | 7.10E+16 | 44.4051 | 44.0033 | 44.4647 |
|  | 2 | -166.175 | 10.891 | 4 | 0.028 | 7.10E+16 | 44.0438 | 43.374 | 44.1431 |
|  | 3 | -8.47405 | 315.4 | 4 | 0 | 6.41641* | 5.61851 | 4.68086 | 5.75754 |
|  | 4 | 382.048 | 781.04 | 4 | 0 | . | -91.5121 | -92.5837 | -91.3532 |
|  | 5 | 391.13 | 18.162 | 4 | 0.001 | . | -93.7824 | -94.854 | -93.6235 |
|  | 6 | 390.597 | -1.0659 | 4 | . | . | -93.6491 | -94.7207 | -93.4903 |
|  | 7 | 388.961 | -3.2709 | 4 | . | . | -93.2403 | -94.3119 | -93.0814 |
|  | 8 | 384.858 | -8.2056 | 4 | . | . | -92.2146 | -93.2862 | -92.0557 |
|  | 9 | 392.056 | 14.396 | 4 | 0.006 | . | -94.014 | -95.0856 | -93.8551 |
|  | 10 | 403.383 | 22.654* | 4 | 0 | . | -96.8458* | -97.9174* | -96.6869* |
| Sweden | 0 | -166.914 |  |  |  | 7.50E+15 | 42.2286 | 42.0946 | 42.2485 |
|  | 1 | -163.031 | 7.7658 | 4 | 0.101 | 8.30E+15 | 42.2579 | 41.856 | 42.3174 |
|  | 2 | -159.289 | 7.4847 | 4 | 0.112 | 1.30E+16 | 42.3223 | 41.6525 | 42.4216 |
|  | 3 | . | . | 4 | . | -1.26794* | . | . | . |
|  | 4 | 391.766 | . | 4 | . | . | -93.9416 | -95.0132 | -93.7827 |
|  | 5 | 390.221 | -3.0903 | 4 | . | . | -93.5553 | -94.6269 | -93.3964 |
|  | 6 | 388.249 | -3.9441 | 4 | . | . | -93.0623 | -94.1339 | -92.9034 |
|  | 7 | 397.363 | 18.227* | 4 | 0.001 | . | -95.3407* | -96.4123* | -95.1818* |
|  | 8 | 390.609 | -13.509 | 4 | . | . | -93.6522 | -94.7238 | -93.4933 |
|  | 9 | 394.17 | 7.1235 | 4 | 0.13 | . | -94.5426 | -95.6142 | -94.3837 |
|  | 10 | 396.755 | 5.1701 | 4 | 0.27 | . | -95.1889 | -96.2605 | -95.03 |
| Switzerland | 0 | -191.94 |  |  |  | 3.90E+18 | 48.4849 | 48.3509 | 48.5047 |
|  | 1 | -188.756 | 6.3668 | 4 | 0.173 | 5.20E+18 | 48.689 | 48.2872 | 48.7486 |
|  | 2 | -179.196 | 19.12 | 4 | 0.001 | 1.80E+18 | 47.2991 | 46.6293 | 47.3984 |
|  | 3 | -32.5627 | 293.27 | 4 | 0 | 2646.58* | 11.6407 | 10.703 | 11.7797 |
|  | 4 | 355.546 | 776.22 | 4 | 0 | . | -84.8864 | -85.958 | -84.7276 |
|  | 5 | 364.018 | 16.944 | 4 | 0.002 | . | -87.0044 | -88.076 | -86.8455 |
|  | 6 | 364.018 | 0 | 4 | . | . | -87.0044 | -88.076 | -86.8455 |
|  | 7 | 353.159 | -21.718 | 4 | . | . | -84.2896 | -85.3613 | -84.1308 |
|  | 8 | 359.586 | 12.855 | 4 | 0.012 | . | -85.8965 | -86.9681 | -85.7376 |
|  | 9 | 358.493 | -2.1857 | 4 | . | . | -85.6233 | -86.6949 | -85.4644 |
|  | 10 | 370.38 | 23.773* | 4 | 0 | . | -88.595* | -89.6666* | -88.4361* |
| Turkey | 0 | -165.82 |  |  |  | 5.70E+15 | 41.955 | 41.8211 | 41.9749 |
|  | 1 | -162.923 | 5.7936 | 4 | 0.215 | 8.10E+15 | 42.2308 | 41.829 | 42.2904 |
|  | 2 | -157.724 | 10.399 | 4 | 0.034 | 8.60E+15 | 41.931 | 41.2612 | 42.0303 |
|  | 3 | -2.73522 | 309.98 | 4 | 0 | 1.52829* | 4.18381 | 3.24615 | 4.32283 |
|  | 4 | 392.446 | 790.36 | 4 | 0 | . | -94.1114 | -95.183 | -93.9525 |
|  | 5 | 395.92 | 6.9487 | 4 | 0.139 | . | -94.98 | -96.0516 | -94.8211 |
|  | 6 | 392.621 | -6.597 | 4 | . | . | -94.1554 | -95.227 | -93.9965 |
|  | 7 | 402.411 | 19.58* | 4 | 0.001 | . | -96.6028 | -97.6744 | -96.4439 |
|  | 8 | 404.033 | 3.2437 | 4 | 0.518 | . | -97.0083 | -98.0799 | -96.8494 |
|  | 9 | 404.033 | 0 | 4 | . | . | -97.0083 | -98.0799 | -96.8494 |
|  | 10 | 404.033 | 0 | 4 | . | . | -97.0083* | -98.0799* | -96.8494* |
| UK | 0 | -191.273 |  |  |  | 3.30E+18 | 48.3181 | 48.1842 | 48.338 |
|  | 1 | -190.666 | 1.2122 | 4 | 0.876 | 8.30E+18 | 49.1666 | 48.7648 | 49.2262 |
|  | 2 | -186.774 | 7.7845 | 4 | 0.1 | 1.20E+19 | 49.1936 | 48.5238 | 49.2929 |
|  | 3 | . | . | 4 | . | -15861.4* | . | . | . |
|  | 4 | 367.619 | . | 4 | . | . | -87.9047 | -88.9763 | -87.7458 |
|  | 5 | 368.12 | 1.0023 | 4 | 0.909 | . | -88.03 | -89.1016 | -87.8711 |
|  | 6 | 378.387 | 20.534* | 4 | 0 | . | -90.5968* | -91.6684* | -90.4379* |
|  | 7 | 375.969 | -4.8366 | 4 | . | . | -89.9922 | -91.0638 | -89.8333 |
|  | 8 | 375.686 | -0.56631 | 4 | . | . | -89.9214 | -90.993 | -89.7625 |
|  | 9 | 375.686 | 0 | 4 | . | . | -89.9214 | -90.993 | -89.7625 |
|  | 10 | 375.686 | 0 | 4 | . | . | -89.9214 | -90.993 | -89.7625 |
| Ukraine | 0 | -151.913 |  |  |  | 1.80E+14 | 38.4782 | 38.3443 | 38.4981 |
|  | 1 | -149.609 | 4.6079 | 4 | 0.33 | 2.90E+14 | 38.9022 | 38.5004 | 38.9618 |
|  | 2 | -144.977 | 9.2628 | 4 | 0.055 | 3.50E+14 | 38.7444 | 38.0746 | 38.8437 |
|  | 3 | 3.77701 | 297.51 | 4 | 0 | .30002* | 2.55575 | 1.61809 | 2.69477 |
|  | 4 | 399.6 | 791.65 | 4 | 0 | . | -95.9001 | -96.9717 | -95.7412 |
|  | 5 | 412.404 | 25.607* | 4 | 0 | . | -99.1009 | -100.173 | -98.9421 |
|  | 6 | 412.855 | 0.90202 | 4 | 0.924 | . | -99.2137* | -100.285* | -99.0548* |
|  | 7 | 407.268 | -11.175 | 4 | . | . | -97.8169 | -98.8885 | -97.658 |
|  | 8 | 398.559 | -17.416 | 4 | . | . | -95.6399 | -96.7115 | -95.481 |
|  | 9 | 403.148 | 9.1781 | 4 | 0.057 | . | -96.7871 | -97.8587 | -96.6282 |
|  | 10 | 407.045 | 7.7927 | 4 | 0.099 | . | -97.7612 | -98.8328 | -97.6023 |

## Table 02: Lag length criteria results for African countries.

|  | **lag** | **LL** | **LR** | **df** | **p** | **FPE** | **AIC** | **HQIC** | **SBIC** |
| --- | --- | --- | --- | --- | --- | --- | --- | --- | --- |
| Angola | 0 | -161.277 |  |  |  | 1.80E+15 | 40.8192 | 40.6853 | 40.8391 |
|  | 1 | -153.304 | 15.947 | 4 | 0.003 | 7.30E+14 | 39.8259 | 39.424 | 39.8855 |
|  | 2 | -141.232 | 24.144 | 4 | 0 | 1.40E+14 | 37.8079 | 37.1381 | 37.9072 |
|  | 3 | 10.5127 | 303.49 | 4 | 0 | .055697* | 0.871816 | -0.06584 | 1.01084 |
|  | 4 | 406.217 | 791.41* | 4 | 0 | . | -97.5541* | -98.6257* | -97.3953* |
|  | 5 | 395.359 | -21.716 | 4 | . | . | -94.8396 | -95.9112 | -94.6807 |
|  | 6 | 395.393 | 0.06975 | 4 | 0.999 | . | -94.8483 | -95.92 | -94.6895 |
|  | 7 | 398.39 | 5.9937 | 4 | 0.2 | . | -95.5976 | -96.6692 | -95.4387 |
|  | 8 | 399.537 | 2.2938 | 4 | 0.682 | . | -95.8843 | -96.9559 | -95.7254 |
|  | 9 | 394.254 | -10.566 | 4 | . | . | -94.5635 | -95.6351 | -94.4046 |
|  | 10 | 390.567 | -7.3736 | 4 | . | . | -93.6418 | -94.7134 | -93.4829 |
| Botswana | 0 | -123.332 |  |  |  | 1.40E+11 | 31.3331 | 31.1991 | 31.3529 |
|  | 1 | -119.288 | 8.0893 | 4 | 0.088 | 1.50E+11 | 31.3219 | 30.9201 | 31.3815 |
|  | 2 | -112.43 | 13.715 | 4 | 0.008 | 1.00E+11 | 30.6075 | 29.9377 | 30.7068 |
|  | 3 | 45.5613 | 315.98 | 4 | 0 | 8.7e-06* | -7.89033 | -8.82798 | -7.75131 |
|  | 4 | 433.002 | 774.88 | 4 | 0 | . | -104.25 | -105.322 | -104.092 |
|  | 5 | 437.621 | 9.2383 | 4 | 0.055 | . | -105.405 | -106.477 | -105.246 |
|  | 6 | 442.277 | 9.3115 | 4 | 0.054 | . | -106.569 | -107.641 | -106.41 |
|  | 7 | 426.045 | -32.463 | 4 | . | . | -102.511 | -103.583 | -102.352 |
|  | 8 | 445.763 | 39.435* | 4 | 0 | . | -107.441* | -108.512* | -107.282* |
|  | 9 | 443.142 | -5.241 | 4 | . | . | -106.786 | -107.857 | -106.627 |
|  | 10 | 443.142 | 0 | 4 | . | . | -106.786 | -107.857 | -106.627 |
| Cabo Verde (Cape Verde) | 0 | -88.6778 |  |  |  | 2.40E+07 | 22.6694 | 22.5355 | 22.6893 |
|  | 1 | -86.1199 | 5.1158 | 4 | 0.276 | 3.70E+07 | 23.03 | 22.6281 | 23.0896 |
|  | 2 | -58.94 | 54.36 | 4 | 0 | 161444 | 17.235 | 16.5652 | 17.3343 |
|  | 3 | . | . | 4 | . | -1.8e-07* | . | . | . |
|  | 4 | 425.164 | . | 4 | . | . | -102.291 | -103.363 | -102.132 |
|  | 5 | 464.552 | 78.776 | 4 | 0 | . | -112.138 | -113.21 | -111.979 |
|  | 6 | 462.783 | -3.5384 | 4 | . | . | -111.696 | -112.767 | -111.537 |
|  | 7 | 475.372 | 25.177 | 4 | 0 | . | -114.843 | -115.915 | -114.684 |
|  | 8 | 484.707 | 18.67* | 4 | 0.001 | . | -117.177 | -118.248 | -117.018 |
|  | 9 | 484.707 | 0 | 4 | . | . | -117.177 | -118.248 | -117.018 |
|  | 10 | 484.707 | 0 | 4 | . | . | -117.177* | -118.248* | -117.018* |
| Djibouti | 0 | -101.555 |  |  |  | 6.00E+08 | 25.8886 | 25.7547 | 25.9085 |
|  | 1 | -98.4234 | 6.2623 | 4 | 0.18 | 8.10E+08 | 26.1059 | 25.704 | 26.1654 |
|  | 2 | -95.2432 | 6.3605 | 4 | 0.174 | 1.40E+09 | 26.3108 | 25.641 | 26.4101 |
|  | 3 | . | . | 4 | . | -9.5e-07* | . | . | . |
|  | 4 | 450.802 | . | 4 | . | . | -108.701* | -109.772* | -108.542* |
|  | 5 | 437.769 | -26.066 | 4 | . | . | -105.442 | -106.514 | -105.283 |
|  | 6 | 442.552 | 9.5665* | 4 | 0.048 | . | -106.638 | -107.71 | -106.479 |
|  | 7 | 442.552 | 0 | 4 | . | . | -106.638 | -107.71 | -106.479 |
|  | 8 | 442.552 | 0 | 4 | . | . | -106.638 | -107.71 | -106.479 |
|  | 9 | 442.552 | 0 | 4 | . | . | -106.638 | -107.71 | -106.479 |
|  | 10 | 442.552 | 0 | 4 | . | . | -106.638 | -107.71 | -106.479 |
| Egypt, Arab Rep. | 0 | -148.758 |  |  |  | 8.00E+13 | 37.6895 | 37.5555 | 37.7094 |
|  | 1 | -148.489 | 0.53719 | 4 | 0.97 | 2.20E+14 | 38.6224 | 38.2205 | 38.6819 |
|  | 2 | -147.172 | 2.6356 | 4 | 0.621 | 6.10E+14 | 39.2929 | 38.6232 | 39.3922 |
|  | 3 | . | . | 4 | . | -.596618* | . | . | . |
|  | 4 | 407.702 | . | 4 | . | . | -97.9255 | -98.9971 | -97.7666 |
|  | 5 | 418.291 | 21.178* | 4 | 0 | . | -100.573 | -101.644 | -100.414 |
|  | 6 | 418.291 | 0 | 4 | . | . | -100.573 | -101.644 | -100.414 |
|  | 7 | 418.291 | 0 | 4 | . | . | -100.573 | -101.644 | -100.414 |
|  | 8 | 420.134 | 3.687 | 4 | 0.45 | . | -101.034 | -102.105 | -100.875 |
|  | 9 | 420.134 | 0 | 4 | . | . | -101.034* | -102.105* | -100.875* |
|  | 10 | 419.064 | -2.1404 | 4 | . | . | -100.766 | -101.838 | -100.607 |
| Eswatini (Swasiland) | 0 | -89.0439 |  |  |  | 2.60E+07 | 22.761 | 22.627 | 22.7808 |
|  | 1 | -86.5536 | 4.9806 | 4 | 0.289 | 4.10E+07 | 23.1384 | 22.7366 | 23.198 |
|  | 2 | -80.997 | 11.113 | 4 | 0.025 | 4.00E+07 | 22.7493 | 22.0795 | 22.8486 |
|  | 3 | . | . | 4 | . | 0* | . | . | . |
|  | 4 | 443.582 | . | 4 | . | . | -106.896 | -107.967 | -106.737 |
|  | 5 | 466.467 | 45.77 | 4 | 0 | . | -112.617 | -113.688 | -112.458 |
|  | 6 | 464.12 | -4.6947 | 4 | . | . | -112.03 | -113.102 | -111.871 |
|  | 7 | 464.12 | 0 | 4 | . | . | -112.03 | -113.102 | -111.871 |
|  | 8 | 467.719 | 7.1993 | 4 | 0.126 | . | -112.93 | -114.001 | -112.771 |
|  | 9 | 468.886 | 2.3333 | 4 | 0.675 | . | -113.221 | -114.293 | -113.063 |
|  | 10 | 475.518 | 13.263* | 4 | 0.01 | . | -114.879* | -115.951* | -114.72* |
| Ethiopia | 0 | -119.08 |  |  |  | 3.60E+12 | 34.5943 | 34.4033 | 34.5789 |
|  | 1 | -117.032 | 4.0972 | 4 | 0.393 | 7.10E+12 | 35.1519 | 34.5788 | 35.1055 |
|  | 2 | -100.673 | 32.718 | 4 | 0 | 3.8e+11* | 31.6208 | 30.6657 | 31.5435 |
|  | 3 | 356.964 | 915.27 | 4 | 0 | . | -97.9897 | -99.3268 | -98.0979 |
|  | 4 | 371.801 | 29.673 | 4 | 0 | . | -102.229 | -103.566 | -102.337 |
|  | 5 | 371.767 | -0.0679 | 4 | . | . | -102.219 | -103.556 | -102.327 |
|  | 6 | 375.577 | 7.6202 | 4 | 0.107 | . | -103.308 | -104.645 | -103.416 |
|  | 7 | 385.959 | 20.765* | 4 | 0 | . | -106.274 | -107.611 | -106.382 |
|  | 8 | 385.959 | 0 | 4 | . | . | -106.274 | -107.611 | -106.382 |
|  | 9 | 385.959 | 0 | 4 | . | . | -106.274 | -107.611 | -106.382 |
|  | 10 | 385.959 | 0 | 4 | . | . | -106.274* | -107.611* | -106.382* |
| Ghana | 0 | -132.312 |  |  |  | 1.30E+12 | 33.578 | 33.444 | 33.5978 |
|  | 1 | -124.847 | 14.93 | 4 | 0.005 | 6.00E+11 | 32.7117 | 32.3099 | 32.7713 |
|  | 2 | -121.511 | 6.6723 | 4 | 0.154 | 1.00E+12 | 32.8777 | 32.2079 | 32.977 |
|  | 3 | . | . | 4 | . | -.000044* | . | . | . |
|  | 4 | 415.192 | . | 4 | . | . | -99.7979 | -100.87 | -99.6391 |
|  | 5 | 430.188 | 29.993 | 4 | 0 | . | -103.547 | -104.619 | -103.388 |
|  | 6 | 433.23 | 6.084 | 4 | 0.193 | . | -104.308 | -105.379 | -104.149 |
|  | 7 | 433.23 | 0 | 4 | . | . | -104.308 | -105.379 | -104.149 |
|  | 8 | 433.23 | 0 | 4 | . | . | -104.308 | -105.379 | -104.149 |
|  | 9 | 438.245 | 10.029* | 4 | 0.04 | . | -105.561 | -106.633 | -105.402 |
|  | 10 | 438.969 | 1.4477 | 4 | 0.836 | . | -105.742* | -106.814* | -105.583* |
| Kenya | 0 | -126.768 |  |  |  | 3.30E+11 | 32.1921 | 32.0582 | 32.212 |
|  | 1 | -124.947 | 3.6422 | 4 | 0.457 | 6.10E+11 | 32.7368 | 32.335 | 32.7964 |
|  | 2 | -119.546 | 10.804 | 4 | 0.029 | 6.10E+11 | 32.3864 | 31.7166 | 32.4857 |
|  | 3 | 32.1483 | 303.39 | 4 | 0 | .000249* | -4.53706 | -5.47472 | -4.39804 |
|  | 4 | 411.83 | 759.36 | 4 | 0 | . | -98.9575 | -100.029 | -98.7986 |
|  | 5 | 432.015 | 40.37* | 4 | 0 | . | -104.004 | -105.075 | -103.845 |
|  | 6 | 432.015 | 0 | 4 | . | . | -104.004* | -105.075* | -103.845* |
|  | 7 | 431.389 | -1.2526 | 4 | . | . | -103.847 | -104.919 | -103.688 |
|  | 8 | 431.389 | 0 | 4 | . | . | -103.847 | -104.919 | -103.688 |
|  | 9 | 431.389 | 0 | 4 | . | . | -103.847 | -104.919 | -103.688 |
|  | 10 | 431.389 | 0 | 4 | . | . | -103.847 | -104.919 | -103.688 |
| Madagascar | 0 | -97.1499 |  |  |  | 6.90E+09 | 28.3285 | 28.1375 | 28.3131 |
|  | 1 | -94.7089 | 4.8819 | 4 | 0.3 | 1.20E+10 | 28.774 | 28.2009 | 28.7276 |
|  | 2 | -77.1099 | 35.198 | 4 | 0 | 4.6e+08* | 24.8885 | 23.9335 | 24.8113 |
|  | 3 | 344.315 | 842.85 | 4 | 0 | . | -94.3758 | -95.7129 | -94.484 |
|  | 4 | 398.967 | 109.3 | 4 | 0 | . | -109.991 | -111.328 | -110.099 |
|  | 5 | 399.986 | 2.0383 | 4 | 0.729 | . | -110.282 | -111.619 | -110.39 |
|  | 6 | 411.436 | 22.901* | 4 | 0 | . | -113.553 | -114.89 | -113.661 |
|  | 7 | 411.58 | 0.28791 | 4 | 0.991 | . | -113.594* | -114.931* | -113.703* |
|  | 8 | 402.831 | -17.499 | 4 | . | . | -111.095 | -112.432 | -111.203 |
|  | 9 | 398.446 | -8.769 | 4 | . | . | -109.842 | -111.179 | -109.95 |
|  | 10 | 395.758 | -5.376 | 4 | . | . | -109.074 | -110.411 | -109.182 |
| Malawi | 0 | -108.063 |  |  |  | 3.10E+09 | 27.5158 | 27.3818 | 27.5356 |
|  | 1 | -103.675 | 8.7762 | 4 | 0.067 | 3.00E+09 | 27.4187 | 27.0169 | 27.4783 |
|  | 2 | -101.921 | 3.5074 | 4 | 0.477 | 7.50E+09 | 27.9803 | 27.3105 | 28.0796 |
|  | 3 | . | . | 4 | . | -2.7e-06* | . | . | . |
|  | 4 | 452.826 | . | 4 | . | . | -109.206 | -110.278 | -109.048 |
|  | 5 | 448.261 | -9.1304 | 4 | . | . | -108.065 | -109.137 | -107.906 |
|  | 6 | 450.886 | 5.2504 | 4 | 0.263 | . | -108.721 | -109.793 | -108.563 |
|  | 7 | 453.507 | 5.243 | 4 | 0.263 | . | -109.377 | -110.448 | -109.218 |
|  | 8 | 453.507 | 0 | 4 | . | . | -109.377 | -110.448 | -109.218 |
|  | 9 | 453.507 | 0 | 4 | . | . | -109.377 | -110.448 | -109.218 |
|  | 10 | 453.507 | 0 | 4 | . | . | -109.377* | -110.448* | -109.218* |
| Mauritius | 0 | -107.818 |  |  |  | 2.90E+09 | 27.4544 | 27.3204 | 27.4742 |
|  | 1 | -102.851 | 9.9323 | 4 | 0.042 | 2.40E+09 | 27.2128 | 26.811 | 27.2724 |
|  | 2 | -99.0805 | 7.5418 | 4 | 0.11 | 3.70E+09 | 27.2701 | 26.6004 | 27.3694 |
|  | 3 | . | . | 4 | . | 0* | . | . | . |
|  | 4 | 453.708 | . | 4 | . | . | -109.427 | -110.499 | -109.268 |
|  | 5 | 451.588 | -4.2398 | 4 | . | . | -108.897 | -109.969 | -108.738 |
|  | 6 | 448.384 | -6.4085 | 4 | . | . | -108.096 | -109.168 | -107.937 |
|  | 7 | 454.642 | 12.517* | 4 | 0.014 | . | -109.661 | -110.732 | -109.502 |
|  | 8 | 454.466 | -0.35269 | 4 | . | . | -109.617 | -110.688 | -109.458 |
|  | 9 | 456.136 | 3.3394 | 4 | 0.503 | . | -110.034 | -111.106 | -109.875 |
|  | 10 | 456.136 | 0 | 4 | . | . | -110.034* | -111.106* | -109.875* |
| Morocco | 0 | -138.409 |  |  |  | 6.00E+12 | 35.1023 | 34.9684 | 35.1222 |
|  | 1 | -136.671 | 3.4761 | 4 | 0.482 | 1.10E+13 | 35.6678 | 35.2659 | 35.7274 |
|  | 2 | -130.669 | 12.004 | 4 | 0.017 | 9.90E+12 | 35.1673 | 34.4976 | 35.2666 |
|  | 3 | . | . | 4 | . | 0* | . | . | . |
|  | 4 | 412.615 | . | 4 | . | . | -99.1537 | -100.225 | -98.9949 |
|  | 5 | 405.972 | -13.285 | 4 | . | . | -97.4931 | -98.5647 | -97.3342 |
|  | 6 | 418.655 | 25.366 | 4 | 0 | . | -100.664 | -101.735 | -100.505 |
|  | 7 | 427.735 | 18.16* | 4 | 0.001 | . | -102.934 | -104.005 | -102.775 |
|  | 8 | 427.735 | 0 | 4 | . | . | -102.934 | -104.005 | -102.775 |
|  | 9 | 427.735 | 0 | 4 | . | . | -102.934 | -104.005 | -102.775 |
|  | 10 | 427.735 | 0 | 4 | . | . | -102.934* | -104.005* | -102.775* |
| Namibia | 0 | -100.306 |  |  |  | 1.70E+10 | 29.2304 | 29.0394 | 29.2149 |
|  | 1 | -98.4509 | 3.7107 | 4 | 0.447 | 3.50E+10 | 29.8431 | 29.2701 | 29.7968 |
|  | 2 | -85.9646 | 24.973 | 4 | 0 | 5.7e+09* | 27.4184 | 26.4634 | 27.3412 |
|  | 3 | 401.553 | 975.03 | 4 | 0 | . | -110.729 | -112.066 | -110.838 |
|  | 4 | 392.034 | -19.037 | 4 | . | . | -108.01 | -109.347 | -108.118 |
|  | 5 | 392.699 | 1.3288 | 4 | 0.856 | . | -108.2 | -109.537 | -108.308 |
|  | 6 | 392.699 | 0 | 4 | . | . | -108.2 | -109.537 | -108.308 |
|  | 7 | 394.915 | 4.4326 | 4 | 0.351 | . | -108.833 | -110.17 | -108.941 |
|  | 8 | 396.027 | 2.2241 | 4 | 0.695 | . | -109.151 | -110.488 | -109.259 |
|  | 9 | 396.027 | 0 | 4 | . | . | -109.151 | -110.488 | -109.259 |
|  | 10 | 406.974 | 21.893* | 4 | 0 | . | -112.278* | -113.615* | -112.386* |
| Nigeria | 0 | -155.203 |  |  |  | 4.00E+14 | 39.3007 | 39.1668 | 39.3206 |
|  | 1 | -154.371 | 1.6639 | 4 | 0.797 | 9.60E+14 | 40.0927 | 39.6909 | 40.1523 |
|  | 2 | -144.767 | 19.209 | 4 | 0.001 | 3.40E+14 | 38.6917 | 38.0219 | 38.791 |
|  | 3 | . | . | 4 | . | 0* | . | . | . |
|  | 4 | 407.708 | . | 4 | . | . | -97.927* | -98.9986* | -97.7681* |
|  | 5 | 402.39 | -10.635 | 4 | . | . | -96.5976 | -97.6692 | -96.4387 |
|  | 6 | 394.578 | -15.626 | 4 | . | . | -94.6444 | -95.716 | -94.4855 |
|  | 7 | 401.578 | 14 | 4 | 0.007 | . | -96.3944 | -97.466 | -96.2355 |
|  | 8 | 406.375 | 9.5953* | 4 | 0.048 | . | -97.5938 | -98.6654 | -97.4349 |
|  | 9 | 406.375 | 0 | 4 | . | . | -97.5938 | -98.6654 | -97.4349 |
|  | 10 | 406.375 | 0 | 4 | . | . | -97.5938 | -98.6654 | -97.4349 |
| Sao Tome and Principe | 0 | -64.3956 |  |  |  | 55591.2 | 16.5989 | 16.465 | 16.6188 |
|  | 1 | -62.8844 | 3.0225 | 4 | 0.554 | 111552 | 17.2211 | 16.8192 | 17.2807 |
|  | 2 | -50.6354 | 24.498 | 4 | 0 | 20247.4 | 15.1589 | 14.4891 | 15.2582 |
|  | 3 | . | . | 4 | . | -2.4e-12* | . | . | . |
|  | 4 | 485.284 | . | 4 | . | . | -117.321 | -118.392 | -117.162 |
|  | 5 | 504.605 | 38.643 | 4 | 0 | . | -122.151* | -123.223* | -121.992* |
|  | 6 | 490.486 | -28.238 | 4 | . | . | -118.622 | -119.693 | -118.463 |
|  | 7 | 491.105 | 1.2374 | 4 | 0.872 | . | -118.776 | -119.848 | -118.617 |
|  | 8 | 491.105 | 0 | 4 | . | . | -118.776 | -119.848 | -118.617 |
|  | 9 | 500.836 | 19.463* | 4 | 0.001 | . | -121.209 | -122.281 | -121.05 |
|  | 10 | 502.736 | 3.7996 | 4 | 0.434 | . | -121.684 | -122.756 | -121.525 |
| Seychelles | 0 | -91.6049 |  |  |  | 5.00E+07 | 23.4012 | 23.2673 | 23.4211 |
|  | 1 | -84.8886 | 13.433 | 4 | 0.009 | 2.70E+07 | 22.7221 | 22.3203 | 22.7817 |
|  | 2 | -75.5641 | 18.649 | 4 | 0.001 | 1.00E+07 | 21.391 | 20.7213 | 21.4903 |
|  | 3 | . | . | 4 | . | 0* | . | . | . |
|  | 4 | 466.52 | . | 4 | . | . | -112.63 | -113.701 | -112.471 |
|  | 5 | 471.784 | 10.528* | 4 | 0.032 | . | -113.946 | -115.018 | -113.787 |
|  | 6 | 473.988 | 4.4095 | 4 | 0.353 | . | -114.497* | -115.569* | -114.338* |
|  | 7 | 472.604 | -2.7697 | 4 | . | . | -114.151 | -115.223 | -113.992 |
|  | 8 | 470.696 | -3.8145 | 4 | . | . | -113.674 | -114.746 | -113.515 |
|  | 9 | 470.696 | 0 | 4 | . | . | -113.674 | -114.746 | -113.515 |
|  | 10 | 470.696 | 0 | 4 | . | . | -113.674 | -114.746 | -113.515 |
| Sierra Leone | 0 | -112.206 |  |  |  | 8.60E+09 | 28.5515 | 28.4175 | 28.5713 |
|  | 1 | -111.834 | 0.74343 | 4 | 0.946 | 2.30E+10 | 29.4586 | 29.0567 | 29.5181 |
|  | 2 | -98.5875 | 26.493 | 4 | 0 | 3.30E+09 | 27.1469 | 26.4771 | 27.2462 |
|  | 3 | . | . | 4 | . | -4.5e-06* | . | . | . |
|  | 4 | 437.004 | . | 4 | . | . | -105.251 | -106.323 | -105.092 |
|  | 5 | 440.024 | 6.0405 | 4 | 0.196 | . | -106.006 | -107.078 | -105.847 |
|  | 6 | 436.08 | -7.889 | 4 | . | . | -105.02 | -106.092 | -104.861 |
|  | 7 | 450.277 | 28.395 | 4 | 0 | . | -108.569 | -109.641 | -108.41 |
|  | 8 | 455.084 | 9.6142* | 4 | 0.047 | . | -109.771 | -110.843 | -109.612 |
|  | 9 | 455.084 | 0 | 4 | . | . | -109.771 | -110.843 | -109.612 |
|  | 10 | 455.084 | 0 | 4 | . | . | -109.771* | -110.843* | -109.612* |
| South Africa | 0 | -132.434 |  |  |  | 1.70E+14 | 38.4097 | 38.2186 | 38.3942 |
|  | 1 | -129.306 | 6.2551 | 4 | 0.181 | 2.40E+14 | 38.6589 | 38.0859 | 38.6126 |
|  | 2 | -91.6202 | 75.372 | 4 | 0 | 2.9e+10* | 29.0343 | 28.0793 | 28.9571 |
|  | 3 | 346.955 | 877.15 | 4 | 0 | . | -95.1301 | -96.4672 | -95.2383 |
|  | 4 | 349.091 | 4.2711 | 4 | 0.371 | . | -95.7402 | -97.0773 | -95.8484 |
|  | 5 | 349.597 | 1.0125 | 4 | 0.908 | . | -95.8849 | -97.222 | -95.9931 |
|  | 6 | 359.41 | 19.627* | 4 | 0.001 | . | -98.6887 | -100.026 | -98.7969 |
|  | 7 | 363.99 | 9.1592 | 4 | 0.057 | . | -99.9971 | -101.334 | -100.105 |
|  | 8 | 363.99 | 0 | 4 | . | . | -99.9971 | -101.334 | -100.105 |
|  | 9 | 363.99 | 0 | 4 | . | . | -99.9971 | -101.334 | -100.105 |
|  | 10 | 363.99 | 0 | 4 | . | . | -99.9971* | -101.334* | -100.105* |
| Tunisia | 0 | -113.694 |  |  |  | 1.30E+10 | 28.9235 | 28.7896 | 28.9434 |
|  | 1 | -103.494 | 20.4 | 4 | 0 | 2.90E+09 | 27.3735 | 26.9716 | 27.4331 |
|  | 2 | -101.836 | 3.3158 | 4 | 0.506 | 7.30E+09 | 27.959 | 27.2893 | 28.0583 |
|  | 3 | . | . | 4 | . | -.00003* | . | . | . |
|  | 4 | 435.559 | . | 4 | . | . | -104.89 | -105.961 | -104.731 |
|  | 5 | 446.581 | 22.044 | 4 | 0 | . | -107.645 | -108.717 | -107.486 |
|  | 6 | 446.581 | 0 | 4 | . | . | -107.645* | -108.717* | -107.486* |
|  | 7 | 440.796 | -11.571 | 4 | . | . | -106.199 | -107.271 | -106.04 |
|  | 8 | 439.665 | -2.2618 | 4 | . | . | -105.916 | -106.988 | -105.757 |
|  | 9 | 445.151 | 10.972* | 4 | 0.027 | . | -107.288 | -108.359 | -107.129 |
|  | 10 | 443.771 | -2.7587 | 4 | . | . | -106.943 | -108.014 | -106.784 |
| Zambia | 0 | -128.454 |  |  |  | 5.00E+11 | 32.6136 | 32.4797 | 32.6335 |
|  | 1 | -126.94 | 3.0294 | 4 | 0.553 | 1.00E+12 | 33.2349 | 32.8331 | 33.2945 |
|  | 2 | -125.943 | 1.9942 | 4 | 0.737 | 3.00E+12 | 33.9857 | 33.3159 | 34.085 |
|  | 3 | . | . | 4 | . | 0* | . | . | . |
|  | 4 | 412.177 | . | 4 | . | . | -99.0444 | -100.116 | -98.8855 |
|  | 5 | 415.887 | 7.4188 | 4 | 0.115 | . | -99.9717 | -101.043 | -99.8128 |
|  | 6 | 446.284 | 60.795* | 4 | 0 | . | -107.571* | -108.643* | -107.412* |
|  | 7 | 440.095 | -12.378 | 4 | . | . | -106.024 | -107.095 | -105.865 |
|  | 8 | 440.095 | 0 | 4 | . | . | -106.024 | -107.095 | -105.865 |
|  | 9 | 440.095 | 0 | 4 | . | . | -106.024 | -107.095 | -105.865 |
|  | 10 | 440.095 | 0 | 4 | . | . | -106.024 | -107.095 | -105.865 |

## Table 03: Lag length criteria results North American countries.

|  | **lag** | **LL** | **LR** | **df** | **p** | **FPE** | **AIC** | **HQIC** | **SBIC** |
| --- | --- | --- | --- | --- | --- | --- | --- | --- | --- |
| Antigua and Barbuda | 0 | -93.43 |  |  |  | 7.90E+07 | 23.8575 | 23.7236 | 23.8774 |
|  | 1 | -92.9516 | 0.95677 | 4 | 0.916 | 2.10E+08 | 24.7379 | 24.3361 | 24.7975 |
|  | 2 | -88.1051 | 9.693 | 4 | 0.046 | 2.40E+08 | 24.5263 | 23.8565 | 24.6256 |
|  | 3 | 70.1373 | 316.48 | 4 | 0 | 1.9e-08* | -14.0343 | -14.972 | -13.8953 |
|  | 4 | 421.069 | 701.86 | 4 | 0 | . | -101.267 | -102.339 | -101.108 |
|  | 5 | 441.21 | 40.281 | 4 | 0 | . | -106.302 | -107.374 | -106.144 |
|  | 6 | 463.56 | 44.701 | 4 | 0 | . | -111.89 | -112.962 | -111.731 |
|  | 7 | 464.454 | 1.7865 | 4 | 0.775 | . | -112.113 | -113.185 | -111.955 |
|  | 8 | 462.95 | -3.0068 | 4 | . | . | -111.738 | -112.809 | -111.579 |
|  | 9 | 470.57 | 15.239* | 4 | 0.004 | . | -113.642* | -114.714* | -113.484* |
|  | 10 | 466.215 | -8.7096 | 4 | . | . | -112.554 | -113.625 | -112.395 |
| Bahamas | 0 | -115.844 |  |  |  | 2.10E+10 | 29.4611 | 29.3271 | 29.4809 |
|  | 1 | -113.605 | 4.478 | 4 | 0.345 | 3.60E+10 | 29.9013 | 29.4995 | 29.9609 |
|  | 2 | -108.516 | 10.177 | 4 | 0.038 | 3.90E+10 | 29.6291 | 28.9594 | 29.7284 |
|  | 3 | 58.6623 | 334.36 | 4 | 0 | 3.3e-07* | -11.1656 | -12.1032 | -11.0266 |
|  | 4 | 436.373 | 755.42 | 4 | 0 | . | -105.093 | -106.165 | -104.934 |
|  | 5 | 424.181 | -24.384 | 4 | . | . | -102.045 | -103.117 | -101.886 |
|  | 6 | 441.732 | 35.102* | 4 | 0 | . | -106.433 | -107.505 | -106.274 |
|  | 7 | 441.732 | 0 | 4 | . | . | -106.433* | -107.505* | -106.274* |
|  | 8 | 439.954 | -3.5563 | 4 | . | . | -105.988 | -107.06 | -105.83 |
|  | 9 | 439.954 | 0 | 4 | . | . | -105.988 | -107.06 | -105.83 |
|  | 10 | 433.628 | -12.651 | 4 | . | . | -104.407 | -105.479 | -104.248 |
| Belize | 0 | -83.0389 |  |  |  | 5.90E+06 | 21.2597 | 21.1258 | 21.2796 |
|  | 1 | -80.3174 | 5.4429 | 4 | 0.245 | 8.70E+06 | 21.5794 | 21.1775 | 21.6389 |
|  | 2 | -68.1976 | 24.24 | 4 | 0 | 1.60E+06 | 19.5494 | 18.8797 | 19.6487 |
|  | 3 | 85.5087 | 307.41 | 4 | 0 | 4.0e-10* | -17.8772 | -18.8148 | -17.7382 |
|  | 4 | 466.175 | 761.33 | 4 | 0 | . | -112.544 | -113.615 | -112.385 |
|  | 5 | 479.334 | 26.318 | 4 | 0 | . | -115.833 | -116.905 | -115.675 |
|  | 6 | 479.037 | -0.59316 | 4 | . | . | -115.759 | -116.831 | -115.6 |
|  | 7 | 487.402 | 16.729 | 4 | 0.002 | . | -117.85 | -118.922 | -117.692 |
|  | 8 | 478.41 | -17.983 | 4 | . | . | -115.603 | -116.674 | -115.444 |
|  | 9 | 483.325 | 9.8292 | 4 | 0.043 | . | -116.831 | -117.903 | -116.672 |
|  | 10 | 489.744 | 12.839* | 4 | 0.012 | . | -118.436* | -119.508* | -118.277* |
| Canada | 0 | -172.981 |  |  |  | 3.40E+16 | 43.7452 | 43.6113 | 43.7651 |
|  | 1 | -171.182 | 3.5968 | 4 | 0.463 | 6.40E+16 | 44.2956 | 43.8938 | 44.3552 |
|  | 2 | -158.527 | 25.31 | 4 | 0 | 1.00E+16 | 42.1318 | 41.4621 | 42.2311 |
|  | 3 | . | . | 4 | . | -2.87089* | . | . | . |
|  | 4 | 384.912 | . | 4 | . | . | -92.2281 | -93.2997 | -92.0692 |
|  | 5 | 381.127 | -7.5714 | 4 | . | . | -91.2816 | -92.3532 | -91.1228 |
|  | 6 | 385.291 | 8.3298 | 4 | 0.08 | . | -92.3229 | -93.3945 | -92.164 |
|  | 7 | 375.114 | -20.354 | 4 | . | . | -89.7786 | -90.8502 | -89.6197 |
|  | 8 | 378.535 | 6.8422 | 4 | 0.144 | . | -90.6339 | -91.7055 | -90.475 |
|  | 9 | 380.688 | 4.3059 | 4 | 0.366 | . | -91.1721 | -92.2437 | -91.0132 |
|  | 10 | 386.683 | 11.99* | 4 | 0.017 | . | -92.6708* | -93.7424* | -92.5119* |
| Costa Rica | 0 | -119.65 |  |  |  | 5.50E+10 | 30.4125 | 30.2785 | 30.4324 |
|  | 1 | -113.731 | 11.839 | 4 | 0.019 | 3.70E+10 | 29.9326 | 29.5308 | 29.9922 |
|  | 2 | -108.985 | 9.4906 | 4 | 0.05 | 4.40E+10 | 29.7463 | 29.0766 | 29.8456 |
|  | 3 | . | . | 4 | . | 0* | . | . | . |
|  | 4 | 437.45 | . | 4 | . | . | -105.362 | -106.434 | -105.204 |
|  | 5 | 433.583 | -7.7336 | 4 | . | . | -104.396 | -105.467 | -104.237 |
|  | 6 | 438.595 | 10.024* | 4 | 0.04 | . | -105.649 | -106.72 | -105.49 |
|  | 7 | 441.356 | 5.5219 | 4 | 0.238 | . | -106.339 | -107.411 | -106.18 |
|  | 8 | 444.758 | 6.8038 | 4 | 0.147 | . | -107.19* | -108.261* | -107.031* |
|  | 9 | 440.304 | -8.9089 | 4 | . | . | -106.076 | -107.148 | -105.917 |
|  | 10 | 437.464 | -5.6787 | 4 | . | . | -105.366 | -106.438 | -105.207 |
| Dominica | 0 | -80.1039 |  |  |  | 2.80E+06 | 20.526 | 20.392 | 20.5458 |
|  | 1 | -74.4261 | 11.355 | 4 | 0.023 | 2.00E+06 | 20.1065 | 19.7047 | 20.1661 |
|  | 2 | -64.1862 | 20.48 | 4 | 0 | 599266 | 18.5465 | 17.8768 | 18.6458 |
|  | 3 | . | . | 4 | . | -5.6e-11* | . | . | . |
|  | 4 | 465.68 | . | 4 | . | . | -112.42 | -113.492 | -112.261 |
|  | 5 | 477.411 | 23.461 | 4 | 0 | . | -115.353 | -116.424 | -115.194 |
|  | 6 | 471.397 | -12.027 | 4 | . | . | -113.849 | -114.921 | -113.69 |
|  | 7 | 483.666 | 24.539* | 4 | 0 | . | -116.917* | -117.988* | -116.758* |
|  | 8 | 477.468 | -12.397 | 4 | . | . | -115.367 | -116.439 | -115.208 |
|  | 9 | 477.179 | -0.57713 | 4 | . | . | -115.295 | -116.366 | -115.136 |
|  | 10 | 477.179 | 0 | 4 | . | . | -115.295 | -116.366 | -115.136 |
| Dominican Republic | 0 | -132.941 |  |  |  | 1.50E+12 | 33.7353 | 33.6013 | 33.7551 |
|  | 1 | -130.402 | 5.0787 | 4 | 0.279 | 2.40E+12 | 34.1004 | 33.6986 | 34.16 |
|  | 2 | -128.962 | 2.8804 | 4 | 0.578 | 6.50E+12 | 34.7404 | 34.0706 | 34.8397 |
|  | 3 | . | . | 4 | . | 0* | . | . | . |
|  | 4 | 422.51 | . | 4 | . | . | -101.627 | -102.699 | -101.469 |
|  | 5 | 435.7 | 26.38 | 4 | 0 | . | -104.925 | -105.996 | -104.766 |
|  | 6 | 440.136 | 8.8736 | 4 | 0.064 | . | -106.034* | -107.106* | -105.875* |
|  | 7 | 428.107 | -24.058 | 4 | . | . | -103.027 | -104.098 | -102.868 |
|  | 8 | 432.925 | 9.6359* | 4 | 0.047 | . | -104.231 | -105.303 | -104.072 |
|  | 9 | 432.379 | -1.0921 | 4 | . | . | -104.095 | -105.166 | -103.936 |
|  | 10 | 431.427 | -1.9044 | 4 | . | . | -103.857 | -104.928 | -103.698 |
| Grenada | 0 | -86.3295 |  |  |  | 1.30E+07 | 22.0824 | 21.9484 | 22.1022 |
|  | 1 | -83.5928 | 5.4735 | 4 | 0.242 | 2.00E+07 | 22.3982 | 21.9963 | 22.4578 |
|  | 2 | -80.8799 | 5.4257 | 4 | 0.246 | 3.90E+07 | 22.72 | 22.0502 | 22.8193 |
|  | 3 | 86.6967 | 335.15 | 4 | 0 | 3.0e-10* | -18.1742 | -19.1118 | -18.0352 |
|  | 4 | 477.419 | 781.45 | 4 | 0 | . | -115.355 | -116.426 | -115.196 |
|  | 5 | 478.214 | 1.5891 | 4 | 0.811 | . | -115.553 | -116.625 | -115.395 |
|  | 6 | 470.441 | -15.547 | 4 | . | . | -113.61 | -114.682 | -113.451 |
|  | 7 | 470.441 | 0 | 4 | . | . | -113.61 | -114.682 | -113.451 |
|  | 8 | 479.463 | 18.045* | 4 | 0.001 | . | -115.866 | -116.937 | -115.707 |
|  | 9 | 479.463 | 0 | 4 | . | . | -115.866 | -116.937 | -115.707 |
|  | 10 | 479.463 | 0 | 4 | . | . | -115.866* | -116.937* | -115.707* |
| Guatemala | 0 | -119.257 |  |  |  | 5.00E+10 | 30.3142 | 30.1802 | 30.3341 |
|  | 1 | -113.769 | 10.976 | 4 | 0.027 | 3.70E+10 | 29.9422 | 29.5404 | 30.0018 |
|  | 2 | -107.893 | 11.752 | 4 | 0.019 | 3.30E+10 | 29.4732 | 28.8035 | 29.5725 |
|  | 3 | . | . | 4 | . | 0* | . | . | . |
|  | 4 | 427.151 | . | 4 | . | . | -102.788 | -103.859 | -102.629 |
|  | 5 | 422.174 | -9.9544 | 4 | . | . | -101.543 | -102.615 | -101.385 |
|  | 6 | 428.168 | 11.989 | 4 | 0.017 | . | -103.042 | -104.114 | -102.883 |
|  | 7 | 429.719 | 3.1004 | 4 | 0.541 | . | -103.43 | -104.501 | -103.271 |
|  | 8 | 439.86 | 20.283 | 4 | 0 | . | -105.965 | -107.037 | -105.806 |
|  | 9 | 439.405 | -0.91086 | 4 | . | . | -105.851 | -106.923 | -105.692 |
|  | 10 | 450.024 | 21.238* | 4 | 0 | . | -108.506* | -109.578* | -108.347* |
| Jamaica | 0 | -110.492 |  |  |  | 5.60E+09 | 28.123 | 27.989 | 28.1428 |
|  | 1 | -107.482 | 6.0193 | 4 | 0.198 | 7.80E+09 | 28.3706 | 27.9687 | 28.4302 |
|  | 2 | -97.865 | 19.235 | 4 | 0.001 | 2.70E+09 | 26.9662 | 26.2965 | 27.0655 |
|  | 3 | 48.2922 | 292.31 | 4 | 0 | 4.4e-06* | -8.57305 | -9.51071 | -8.43403 |
|  | 4 | 451.985 | 807.39 | 4 | 0 | . | -108.996 | -110.068 | -108.837 |
|  | 5 | 446.942 | -10.086 | 4 | . | . | -107.736 | -108.807 | -107.577 |
|  | 6 | 429.403 | -35.08 | 4 | . | . | -103.351 | -104.422 | -103.192 |
|  | 7 | 439.098 | 19.391 | 4 | 0.001 | . | -105.775 | -106.846 | -105.616 |
|  | 8 | 445.226 | 12.255 | 4 | 0.016 | . | -107.306 | -108.378 | -107.147 |
|  | 9 | 442.816 | -4.8193 | 4 | . | . | -106.704 | -107.776 | -106.545 |
|  | 10 | 462.599 | 39.566* | 4 | 0 | . | -111.65* | -112.721* | -111.491* |
| Mexico | 0 | -141.931 |  |  |  | 2.5e+15* | 41.1231 | 40.9321 | 41.1076 |
|  | 1 | -138.396 | 7.0702 | 4 | 0.132 | 3.20E+15 | 41.2559 | 40.6829 | 41.2095 |
|  | 2 | -133.137 | 10.517 | 4 | 0.033 | 4.10E+15 | 40.8964 | 39.9413 | 40.8191 |
|  | 3 | 349.599 | 965.47* | 4 | 0 | . | -95.8856* | -97.2226* | -95.9937* |
|  | 4 | 349.513 | -0.17285 | 4 | . | . | -95.8609 | -97.1979 | -95.969 |
|  | 5 | . | . | 4 | . | . | . | . | . |
|  | 6 | 342.839 | . | 4 | . | . | -93.954 | -95.2911 | -94.0622 |
|  | 7 | 342.839 | 0 | 4 | . | . | -93.954 | -95.2911 | -94.0622 |
|  | 8 | 342.839 | 0 | 4 | . | . | -93.954 | -95.2911 | -94.0622 |
|  | 9 | 342.839 | 0 | 4 | . | . | -93.954 | -95.2911 | -94.0622 |
|  | 10 | 342.839 | 0 | 4 | . | . | -93.954 | -95.2911 | -94.0622 |
| Nicaragua | 0 | -110.321 |  |  |  | 5.40E+09 | 28.0803 | 27.9463 | 28.1002 |
|  | 1 | -104.982 | 10.678 | 4 | 0.03 | 4.20E+09 | 27.7455 | 27.3437 | 27.8051 |
|  | 2 | -97.3697 | 15.225 | 4 | 0.004 | 2.40E+09 | 26.8424 | 26.1727 | 26.9417 |
|  | 3 | . | . | 4 | . | -3.8e-06* | . | . | . |
|  | 4 | 450.943 | . | 4 | . | . | -108.736 | -109.807 | -108.577 |
|  | 5 | 443.268 | -15.35 | 4 | . | . | -106.817 | -107.889 | -106.658 |
|  | 6 | 446.672 | 6.8069 | 4 | 0.146 | . | -107.668 | -108.739 | -107.509 |
|  | 7 | 455.447 | 17.551 | 4 | 0.002 | . | -109.862 | -110.933 | -109.703 |
|  | 8 | 450.271 | -10.352 | 4 | . | . | -108.568 | -109.639 | -108.409 |
|  | 9 | 459.58 | 18.618* | 4 | 0.001 | . | -110.895* | -111.967* | -110.736* |
|  | 10 | 457.819 | -3.5219 | 4 | . | . | -110.455 | -111.526 | -110.296 |
| Panama | 0 | -119.412 |  |  |  | 4.00E+12 | 34.6891 | 34.4981 | 34.6736 |
|  | 1 | -118.866 | 1.0909 | 4 | 0.896 | 1.20E+13 | 35.6761 | 35.1031 | 35.6297 |
|  | 2 | -100.049 | 37.634 | 4 | 0 | 3.2e+11* | 31.4427 | 30.4876 | 31.3654 |
|  | 3 | 334.951 | 870 | 4 | 0 | . | -91.7003 | -93.0374 | -91.8085 |
|  | 4 | 343.979 | 18.056 | 4 | 0.001 | . | -94.2798 | -95.6169 | -94.388 |
|  | 5 | 360.575 | 33.191 | 4 | 0 | . | -99.0214 | -100.358 | -99.1296 |
|  | 6 | 357.038 | -7.073 | 4 | . | . | -98.0109 | -99.348 | -98.1191 |
|  | 7 | 359.419 | 4.7605 | 4 | 0.313 | . | -98.691 | -100.028 | -98.7992 |
|  | 8 | 372.64 | 26.444 | 4 | 0 | . | -102.469* | -103.806* | -102.577* |
|  | 9 | 360.234 | -24.814 | 4 | . | . | -98.9239 | -100.261 | -99.032 |
|  | 10 | 368.374 | 16.281* | 4 | 0.003 | . | -101.25 | -102.587 | -101.358 |
| St. Lucia | 0 | -95.1832 |  |  |  | 1.20E+08 | 24.2958 | 24.1619 | 24.3157 |
|  | 1 | -90.2884 | 9.7897 | 4 | 0.044 | 1.10E+08 | 24.0721 | 23.6702 | 24.1317 |
|  | 2 | -87.3005 | 5.9757 | 4 | 0.201 | 1.90E+08 | 24.3251 | 23.6554 | 24.4244 |
|  | 3 | 73.6829 | 321.97 | 4 | 0 | 7.7e-09* | -14.9207 | -15.8584 | -14.7817 |
|  | 4 | 461.518 | 775.67 | 4 | 0 | . | -111.38 | -112.451 | -111.221 |
|  | 5 | 466.22 | 9.4032 | 4 | 0.052 | . | -112.555 | -113.627 | -112.396 |
|  | 6 | 458.08 | -16.281 | 4 | . | . | -110.52 | -111.592 | -110.361 |
|  | 7 | 466.356 | 16.552 | 4 | 0.002 | . | -112.589 | -113.661 | -112.43 |
|  | 8 | 469.185 | 5.6588 | 4 | 0.226 | . | -113.296 | -114.368 | -113.137 |
|  | 9 | 478.121 | 17.873* | 4 | 0.001 | . | -115.53* | -116.602* | -115.371* |
|  | 10 | 473.924 | -8.3943 | 4 | . | . | -114.481 | -115.553 | -114.322 |
| St. Vincent and the Grenadines | 0 | -81.6419 |  |  |  | 4.10E+06 | 20.9105 | 20.7765 | 20.9303 |
|  | 1 | -80.7479 | 1.788 | 4 | 0.775 | 9.70E+06 | 21.687 | 21.2851 | 21.7466 |
|  | 2 | -65.7223 | 30.051 | 4 | 0 | 879850 | 18.9306 | 18.2608 | 19.0299 |
|  | 3 | 104.048 | 339.54 | 4 | 0 | 3.9e-12* | -22.512 | -23.4497 | -22.373 |
|  | 4 | 460.983 | 713.87 | 4 | 0 | . | -111.246 | -112.317 | -111.087 |
|  | 5 | 478.647 | 35.327* | 4 | 0 | . | -115.662 | -116.733 | -115.503 |
|  | 6 | 479.243 | 1.1925 | 4 | 0.879 | . | -115.811 | -116.882 | -115.652 |
|  | 7 | 483.763 | 9.0406 | 4 | 0.06 | . | -116.941 | -118.012 | -116.782 |
|  | 8 | 483.763 | 0 | 4 | . | . | -116.941* | -118.012* | -116.782* |
|  | 9 | 478.218 | -11.09 | 4 | . | . | -115.555 | -116.626 | -115.396 |
|  | 10 | 479.289 | 2.1416 | 4 | 0.71 | . | -115.822 | -116.894 | -115.663 |
| Trinidad and Tobago | 0 | -135.777 |  |  |  | 3.10E+12 | 34.4443 | 34.3104 | 34.4642 |
|  | 1 | -130.618 | 10.319 | 4 | 0.035 | 2.50E+12 | 34.1544 | 33.7526 | 34.214 |
|  | 2 | -114.622 | 31.992 | 4 | 0 | 1.80E+11 | 31.1555 | 30.4857 | 31.2548 |
|  | 3 | 25.7709 | 280.79 | 4 | 0 | .001228* | -2.94273 | -3.88038 | -2.80371 |
|  | 4 | 420.35 | 789.16 | 4 | 0 | . | -101.088 | -102.159 | -100.929 |
|  | 5 | 419.603 | -1.4943 | 4 | . | . | -100.901 | -101.972 | -100.742 |
|  | 6 | 417.256 | -4.6934 | 4 | . | . | -100.314 | -101.386 | -100.155 |
|  | 7 | 424.449 | 14.386* | 4 | 0.006 | . | -102.112* | -103.184* | -101.953* |
|  | 8 | 417.631 | -13.636 | 4 | . | . | -100.408 | -101.479 | -100.249 |
|  | 9 | 422.124 | 8.9854 | 4 | 0.061 | . | -101.531 | -102.603 | -101.372 |
|  | 10 | 423.757 | 3.2661 | 4 | 0.514 | . | -101.939 | -103.011 | -101.78 |
| USA | 0 | -201.361 |  |  |  | 4.10E+19 | 50.8401 | 50.7062 | 50.86 |
|  | 1 | -199.853 | 3.0149 | 4 | 0.555 | 8.30E+19 | 51.4633 | 51.0614 | 51.5229 |
|  | 2 | -194.717 | 10.272 | 4 | 0.036 | 8.90E+19 | 51.1793 | 50.5095 | 51.2786 |
|  | 3 | . | . | 4 | . | 0* | . | . | . |
|  | 4 | 360.707 | . | 4 | . | . | -86.1766 | -87.2482 | -86.0177 |
|  | 5 | 362.687 | 3.9606 | 4 | 0.411 | . | -86.6717 | -87.7433 | -86.5128 |
|  | 6 | 377.7 | 30.027 | 4 | 0 | . | -90.4251* | -91.4967* | -90.2662* |
|  | 7 | 368.986 | -17.428 | 4 | . | . | -88.2466 | -89.3182 | -88.0877 |
|  | 8 | 367.598 | -2.7776 | 4 | . | . | -87.8994 | -88.971 | -87.7405 |
|  | 9 | 359.672 | -15.852 | 4 | . | . | -85.9179 | -86.9895 | -85.759 |
|  | 10 | 369.63 | 19.916* | 4 | 0.001 | . | -88.4074 | -89.479 | -88.2486 |

## Table 04: Lag length criteria results for South American countries.

|  | **lag** | **LL** | **LR** | **df** | **p** | **FPE** | **AIC** | **HQIC** | **SBIC** |
| --- | --- | --- | --- | --- | --- | --- | --- | --- | --- |
| Argentina | 0 | -161.174 |  |  |  | 1.80E+15 | 40.7935 | 40.6596 | 40.8134 |
|  | 1 | -160.297 | 1.7537 | 4 | 0.781 | 4.20E+15 | 41.5743 | 41.1725 | 41.6339 |
|  | 2 | -157.609 | 5.3761 | 4 | 0.251 | 8.30E+15 | 41.9023 | 41.2326 | 42.0016 |
|  | 3 | -7.52774 | 300.16 | 4 | 0 | 5.06463* | 5.38194 | 4.44428 | 5.52096 |
|  | 4 | 379.668 | 774.39 | 4 | 0 | . | -90.917 | -91.9886 | -90.7581 |
|  | 5 | 401.486 | 43.635 | 4 | 0 | . | -96.3714 | -97.443 | -96.2125 |
|  | 6 | 406.978 | 10.985* | 4 | 0.027 | . | -97.7445 | -98.8161 | -97.5856 |
|  | 7 | 406.978 | 0 | 4 | . | . | -97.7445 | -98.8161 | -97.5856 |
|  | 8 | 406.978 | 0 | 4 | . | . | -97.7445 | -98.8161 | -97.5856 |
|  | 9 | 406.978 | 0 | 4 | . | . | -97.7445 | -98.8161 | -97.5856 |
|  | 10 | 406.978 | 0 | 4 | . | . | -97.7445* | -98.8161* | -97.5856* |
| Bolivia | 0 | -129.349 |  |  |  | 6.30E+11 | 32.8373 | 32.7034 | 32.8572 |
|  | 1 | -128.176 | 2.3476 | 4 | 0.672 | 1.40E+12 | 33.5439 | 33.142 | 33.6035 |
|  | 2 | -120.045 | 16.261 | 4 | 0.003 | 7.00E+11 | 32.5112 | 31.8415 | 32.6105 |
|  | 3 | . | . | 4 | . | -.00003* | . | . | . |
|  | 4 | 420.677 | . | 4 | . | . | -101.169 | -102.241 | -101.01 |
|  | 5 | 435.89 | 30.427* | 4 | 0 | . | -104.973 | -106.044 | -104.814 |
|  | 6 | 435.89 | 0 | 4 | . | . | -104.973 | -106.044 | -104.814 |
|  | 7 | 435.89 | 0 | 4 | . | . | -104.973 | -106.044 | -104.814 |
|  | 8 | 435.89 | 0 | 4 | . | . | -104.973 | -106.044 | -104.814 |
|  | 9 | 435.89 | 0 | 4 | . | . | -104.973 | -106.044 | -104.814 |
|  | 10 | 435.89 | 0 | 4 | . | . | -104.973* | -106.044* | -104.814* |
| Brazil | 0 | -156.658 |  |  |  | 1.7e+17* | 45.3308 | 45.1398 | 45.3154 |
|  | 1 | -154.201 | 4.9144 | 4 | 0.296 | 2.90E+17 | 45.7716 | 45.1986 | 45.7253 |
|  | 2 | -149.38 | 9.6422 | 4 | 0.047 | 4.20E+17 | 45.537 | 44.582 | 45.4597 |
|  | 3 | 332.01 | 962.78 | 4 | 0 | . | -90.86 | -92.1971 | -90.9682 |
|  | 4 | 332.42 | 0.82071 | 4 | 0.936 | . | -90.9773 | -92.3144 | -91.0855 |
|  | 5 | 329.632 | -5.5766 | 4 | . | . | -90.1806 | -91.5177 | -90.2888 |
|  | 6 | 338.078 | 16.892 | 4 | 0.002 | . | -92.5937 | -93.9308 | -92.7019 |
|  | 7 | 339.316 | 2.4753 | 4 | 0.649 | . | -92.9473 | -94.2844 | -93.0555 |
|  | 8 | 332.492 | -13.646 | 4 | . | . | -90.9978 | -92.3349 | -91.106 |
|  | 9 | 332.492 | 0 | 4 | . | . | -90.9978 | -92.3349 | -91.106 |
|  | 10 | 339.744 | 14.504* | 4 | 0.006 | . | -93.0698* | -94.4069* | -93.178* |
| Chile | 0 | -159.694 |  |  |  | 1.20E+15 | 40.4234 | 40.2895 | 40.4433 |
|  | 1 | -159.233 | 0.92116 | 4 | 0.922 | 3.20E+15 | 41.3083 | 40.9064 | 41.3678 |
|  | 2 | -152.472 | 13.523 | 4 | 0.009 | 2.30E+15 | 40.6179 | 39.9481 | 40.7172 |
|  | 3 | . | . | 4 | . | 0* | . | . | . |
|  | 4 | 393.467 | . | 4 | . | . | -94.3668 | -95.4384 | -94.2079 |
|  | 5 | 408.294 | 29.653 | 4 | 0 | . | -98.0735 | -99.1451 | -97.9146 |
|  | 6 | 406.997 | -2.594 | 4 | . | . | -97.7492 | -98.8208 | -97.5903 |
|  | 7 | 401.951 | -10.093 | 4 | . | . | -96.4876 | -97.5592 | -96.3287 |
|  | 8 | 411.793 | 19.684* | 4 | 0.001 | . | -98.9481* | -100.02* | -98.7892* |
|  | 9 | 410.109 | -3.3664 | 4 | . | . | -98.5273 | -99.5989 | -98.3684 |
|  | 10 | 402.237 | -15.744 | 4 | . | . | -96.5593 | -97.6309 | -96.4005 |
| Colombia | 0 | -154.652 |  |  |  | 3.50E+14 | 39.163 | 39.0291 | 39.1829 |
|  | 1 | -150.709 | 7.8859 | 4 | 0.096 | 3.80E+14 | 39.1773 | 38.7754 | 39.2369 |
|  | 2 | -147.116 | 7.186 | 4 | 0.126 | 6.00E+14 | 39.279 | 38.6093 | 39.3783 |
|  | 3 | . | . | 4 | . | -.017443* | . | . | . |
|  | 4 | 388.684 | . | 4 | . | . | -93.171 | -94.2426 | -93.0121 |
|  | 5 | 404.909 | 32.449 | 4 | 0 | . | -97.2272 | -98.2988 | -97.0683 |
|  | 6 | 410.415 | 11.012* | 4 | 0.026 | . | -98.6037* | -99.6753* | -98.4448* |
|  | 7 | 406.98 | -6.8704 | 4 | . | . | -97.7449 | -98.8165 | -97.586 |
|  | 8 | 408.468 | 2.9762 | 4 | 0.562 | . | -98.1169 | -99.1885 | -97.958 |
|  | 9 | 408.468 | 0 | 4 | . | . | -98.1169 | -99.1885 | -97.958 |
|  | 10 | 408.468 | 0 | 4 | . | . | -98.1169 | -99.1885 | -97.958 |
| Ecuador | 0 | -125.875 |  |  |  | 2.60E+11 | 31.9688 | 31.8349 | 31.9887 |
|  | 1 | -121.618 | 8.5152 | 4 | 0.074 | 2.70E+11 | 31.9044 | 31.5026 | 31.964 |
|  | 2 | -112.852 | 17.532 | 4 | 0.002 | 1.20E+11 | 30.7129 | 30.0432 | 30.8122 |
|  | 3 | 34.6527 | 295.01 | 4 | 0 | .000133* | -5.16316 | -6.10082 | -5.02414 |
|  | 4 | 421.653 | 774 | 4 | 0 | . | -101.413 | -102.485 | -101.254 |
|  | 5 | 437.855 | 32.404 | 4 | 0 | . | -105.464 | -106.535 | -105.305 |
|  | 6 | 428.279 | -19.152 | 4 | . | . | -103.07 | -104.141 | -102.911 |
|  | 7 | 428.41 | 0.26145 | 4 | 0.992 | . | -103.102 | -104.174 | -102.944 |
|  | 8 | 440.565 | 24.311* | 4 | 0 | . | -106.141 | -107.213 | -105.982 |
|  | 9 | 440.565 | 0 | 4 | . | . | -106.141 | -107.213 | -105.982 |
|  | 10 | 440.565 | 0 | 4 | . | . | -106.141* | -107.213* | -105.982* |
| Guyana | 0 | -97.458 |  |  |  | 8.60E+11 | 33.1527 | 32.8748 | 33.0833 |
|  | 1 | -87.4691 | 19.978 | 4 | 0.001 | 1.40E+11 | 31.1564 | 30.3228 | 30.9481 |
|  | 2 | . | . | 4 | . | -9.5e-06* | . | . | . |
|  | 3 | 316.249 | . | 4 | . | . | -101.416 | -103.084 | -101.833 |
|  | 4 | 319.135 | 5.7729 | 4 | 0.217 | . | -102.378 | -104.046 | -102.795 |
|  | 5 | 319.135 | 0 | 4 | . | . | -102.378 | -104.046 | -102.795 |
|  | 6 | 319.135 | 0 | 4 | . | . | -102.378 | -104.046 | -102.795 |
|  | 7 | 327.339 | 16.407* | 4 | 0.003 | . | -105.113 | -106.78 | -105.529 |
|  | 8 | 327.339 | 0 | 4 | . | . | -105.113 | -106.78 | -105.529 |
|  | 9 | 327.339 | 0 | 4 | . | . | -105.113 | -106.78 | -105.529 |
|  | 10 | 327.339 | 0 | 4 | . | . | -105.113* | -106.78* | -105.529* |
| Paraguay | 0 | -119.298 |  |  |  | 5.10E+10 | 30.3245 | 30.1905 | 30.3443 |
|  | 1 | -114.885 | 8.8254 | 4 | 0.066 | 4.90E+10 | 30.2213 | 29.8195 | 30.2809 |
|  | 2 | -105.559 | 18.652* | 4 | 0.001 | 1.90E+10 | 28.8898 | 28.22 | 28.9891 |
|  | 3 | . | . | 4 | . | -3.6e-06* | . | . | . |
|  | 4 | 443.505 | . | 4 | . | . | -106.876 | -107.948 | -106.717 |
|  | 5 | 443.505 | 0 | 4 | . | . | -106.876* | -107.948* | -106.717* |
|  | 6 | 440.966 | -5.0768 | 4 | . | . | -106.242 | -107.313 | -106.083 |
|  | 7 | 440.966 | 0 | 4 | . | . | -106.242 | -107.313 | -106.083 |
|  | 8 | 440.966 | 0 | 4 | . | . | -106.242 | -107.313 | -106.083 |
|  | 9 | 440.966 | 0 | 4 | . | . | -106.242 | -107.313 | -106.083 |
|  | 10 | 440.966 | 0 | 4 | . | . | -106.242 | -107.313 | -106.083 |
| Peru | 0 | -149.491 |  |  |  | 9.60E+13 | 37.8728 | 37.7389 | 37.8927 |
|  | 1 | -147.15 | 4.683 | 4 | 0.321 | 1.60E+14 | 38.2875 | 37.8856 | 38.347 |
|  | 2 | -141.382 | 11.535 | 4 | 0.021 | 1.40E+14 | 37.8456 | 37.1758 | 37.9449 |
|  | 3 | . | . | 4 | . | -.017433* | . | . | . |
|  | 4 | 386.011 | . | 4 | . | . | -92.5027 | -93.5743 | -92.3438 |
|  | 5 | 401.188 | 30.355 | 4 | 0 | . | -96.2971 | -97.3687 | -96.1382 |
|  | 6 | 413.449 | 24.521* | 4 | 0 | . | -99.3622 | -100.434 | -99.2033 |
|  | 7 | 417.411 | 7.9242 | 4 | 0.094 | . | -100.353 | -101.424 | -100.194 |
|  | 8 | 417.411 | 0 | 4 | . | . | -100.353 | -101.424 | -100.194 |
|  | 9 | 417.411 | 0 | 4 | . | . | -100.353 | -101.424 | -100.194 |
|  | 10 | 417.411 | 0 | 4 | . | . | -100.353* | -101.424* | -100.194* |
| Uruguay | 0 | -132.581 |  |  |  | 1.40E+12 | 33.6453 | 33.5113 | 33.6651 |
|  | 1 | -131.303 | 2.5563 | 4 | 0.635 | 3.00E+12 | 34.3257 | 33.9239 | 34.3853 |
|  | 2 | -130.537 | 1.5316 | 4 | 0.821 | 9.60E+12 | 35.1343 | 34.4645 | 35.2336 |
|  | 3 | 21.733 | 304.54 | 4 | 0 | .00337* | -1.93325 | -2.87091 | -1.79423 |
|  | 4 | 421.995 | 800.52 | 4 | 0 | . | -101.499 | -102.57 | -101.34 |
|  | 5 | 421.397 | -1.1969 | 4 | . | . | -101.349 | -102.421 | -101.19 |
|  | 6 | 418.225 | -6.3436 | 4 | . | . | -100.556 | -101.628 | -100.397 |
|  | 7 | 415.263 | -5.9252 | 4 | . | . | -99.8156 | -100.887 | -99.6568 |
|  | 8 | 432.923 | 35.321* | 4 | 0 | . | -104.231 | -105.302 | -104.072 |
|  | 9 | 432.923 | 0 | 4 | . | . | -104.231 | -105.302 | -104.072 |
|  | 10 | 432.923 | 0 | 4 | . | . | -104.231* | -105.302* | -104.072* |

## Table 05: Lag length criteria results for Asia and Oceania countries.

| **Country** | **lag** | **LL** | **LR** | **df** | **p** | **FPE** | **AIC** | **HQIC** | **SBIC** |
| --- | --- | --- | --- | --- | --- | --- | --- | --- | --- |
| Armenia | 0 | -109.243 |  |  |  | 4.10E+09 | 27.8107 | 27.6767 | 27.8306 |
|  | 1 | -104.587 | 9.3122 | 4 | 0.054 | 3.80E+09 | 27.6467 | 27.2448 | 27.7063 |
|  | 2 | -97.4546 | 14.264 | 4 | 0.006 | 2.50E+09 | 26.8636 | 26.1939 | 26.9629 |
|  | 3 | . | . | 4 | . | -4.2e-08* | . | . | . |
|  | 4 | 459.692 | . | 4 | . | . | -110.923 | -111.995 | -110.764 |
|  | 5 | 448.166 | -23.053 | 4 | . | . | -108.042 | -109.113 | -107.883 |
|  | 6 | 459.076 | 21.82 | 4 | 0 | . | -110.769 | -111.841 | -110.61 |
|  | 7 | 457.1 | -3.9518 | 4 | . | . | -110.275 | -111.347 | -110.116 |
|  | 8 | 462.271 | 10.341* | 4 | 0.035 | . | -111.568 | -112.639 | -111.409 |
|  | 9 | 461.766 | -1.0093 | 4 | . | . | -111.442 | -112.513 | -111.283 |
|  | 10 | 464.567 | 5.6025 | 4 | 0.231 | . | -112.142* | -113.213* | -111.983* |
| Australia | 0 | -174.396 |  |  |  | 4.90E+16 | 44.0989 | 43.9649 | 44.1187 |
|  | 1 | -173.452 | 1.8879 | 4 | 0.756 | 1.10E+17 | 44.8629 | 44.461 | 44.9225 |
|  | 2 | -167.443 | 12.018 | 4 | 0.017 | 9.70E+16 | 44.3606 | 43.6909 | 44.4599 |
|  | 3 | 1.30232 | 337.49 | 4 | 0 | .556975* | 3.17442 | 2.23677 | 3.31344 |
|  | 4 | 360.074 | 717.54 | 4 | 0 | . | -86.0184 | -87.09 | -85.8595 |
|  | 5 | 377.557 | 34.967 | 4 | 0 | . | -90.3893 | -91.4609 | -90.2304 |
|  | 6 | 385.746 | 16.378* | 4 | 0.003 | . | -92.4366 | -93.5082 | -92.2777 |
|  | 7 | 389.758 | 8.0238 | 4 | 0.091 | . | -93.4396 | -94.5112 | -93.2807 |
|  | 8 | 389.609 | -0.29947 | 4 | . | . | -93.4022 | -94.4738 | -93.2433 |
|  | 9 | 393.123 | 7.0293 | 4 | 0.134 | . | -94.2808* | -95.3524* | -94.1219* |
|  | 10 | 383.049 | -20.149 | 4 | . | . | -91.7622 | -92.8338 | -91.6033 |
| Azerbaijan | 0 | -147.433 |  |  |  | 5.80E+13 | 37.3582 | 37.2243 | 37.3781 |
|  | 1 | -139.896 | 15.074 | 4 | 0.005 | 2.60E+13 | 36.474 | 36.0721 | 36.5336 |
|  | 2 | -128.938 | 21.916 | 4 | 0 | 6.40E+12 | 34.7345 | 34.0647 | 34.8338 |
|  | 3 | 34.7946 | 327.46 | 4 | 0 | .000129* | -5.19865 | -6.1363 | -5.05963 |
|  | 4 | 407.816 | 746.04 | 4 | 0 | . | -97.954 | -99.0256 | -97.7951 |
|  | 5 | 411.827 | 8.023 | 4 | 0.091 | . | -98.9569 | -100.028 | -98.798 |
|  | 6 | 422.22 | 20.784 | 4 | 0 | . | -101.555 | -102.627 | -101.396 |
|  | 7 | 434.221 | 24.002 | 4 | 0 | . | -104.555* | -105.627* | -104.396* |
|  | 8 | 418.777 | -30.888 | 4 | . | . | -100.694 | -101.766 | -100.535 |
|  | 9 | 427.459 | 17.364* | 4 | 0.002 | . | -102.865 | -103.936 | -102.706 |
|  | 10 | 425.421 | -4.0756 | 4 | . | . | -102.355 | -103.427 | -102.196 |
| Bangaladesh | 0 | -137.753 |  |  |  | 5.10E+12 | 34.9383 | 34.8044 | 34.9582 |
|  | 1 | -136.462 | 2.5827 | 4 | 0.63 | 1.10E+13 | 35.6155 | 35.2136 | 35.6751 |
|  | 2 | -128.781 | 15.362 | 4 | 0.004 | 6.20E+12 | 34.6953 | 34.0255 | 34.7946 |
|  | 3 | . | . | 4 | . | 0* | . | . | . |
|  | 4 | 416.898 | . | 4 | . | . | -100.224 | -101.296 | -100.066 |
|  | 5 | 412.241 | -9.3129 | 4 | . | . | -99.0603 | -100.132 | -98.9014 |
|  | 6 | 419.119 | 13.755* | 4 | 0.008 | . | -100.78 | -101.851 | -100.621 |
|  | 7 | 419.119 | 0 | 4 | . | . | -100.78 | -101.851 | -100.621 |
|  | 8 | 419.119 | 0 | 4 | . | . | -100.78* | -101.851* | -100.621* |
|  | 9 | 418.977 | -0.28311 | 4 | . | . | -100.744 | -101.816 | -100.585 |
|  | 10 | 416.066 | -5.8214 | 4 | . | . | -100.017 | -101.088 | -99.8577 |
| Cambodia | 0 | -120.021 |  |  |  | 6.10E+10 | 30.5052 | 30.3712 | 30.5251 |
|  | 1 | -119.454 | 1.1335 | 4 | 0.889 | 1.50E+11 | 31.3635 | 30.9617 | 31.4231 |
|  | 2 | -105.059 | 28.79 | 4 | 0 | 1.60E+10 | 28.7647 | 28.095 | 28.864 |
|  | 3 | . | . | 4 | . | -.000014* | . | . | . |
|  | 4 | 400.977 | . | 4 | . | . | -96.2442 | -97.3158 | -96.0853 |
|  | 5 | 442.916 | 83.879 | 4 | 0 | . | -106.729 | -107.801 | -106.57 |
|  | 6 | 442.916 | 0 | 4 | . | . | -106.729 | -107.801 | -106.57 |
|  | 7 | 450.046 | 14.26 | 4 | 0.007 | . | -108.511 | -109.583 | -108.353 |
|  | 8 | 450.046 | 0 | 4 | . | . | -108.511* | -109.583* | -108.353* |
|  | 9 | 440.329 | -19.434 | 4 | . | . | -106.082 | -107.154 | -105.923 |
|  | 10 | 449.904 | 19.15* | 4 | 0.001 | . | -108.476 | -109.548 | -108.317 |
| China | 0 | -201.308 |  |  |  | 4.10E+19 | 50.827 | 50.6931 | 50.8469 |
|  | 1 | -200.57 | 1.4754 | 4 | 0.831 | 9.90E+19 | 51.6426 | 51.2407 | 51.7022 |
|  | 2 | -189.981 | 21.18 | 4 | 0 | 2.70E+19 | 49.9951 | 49.3254 | 50.0944 |
|  | 3 | . | . | 4 | . | 0* | . | . | . |
|  | 4 | 368.27 | . | 4 | . | . | -88.0676 | -89.1392 | -87.9087 |
|  | 5 | 360.355 | -15.831 | 4 | . | . | -86.0887 | -87.1603 | -85.9298 |
|  | 6 | 371.879 | 23.05* | 4 | 0 | . | -88.9699 | -90.0415 | -88.811 |
|  | 7 | 371.879 | 0 | 4 | . | . | -88.9699 | -90.0415 | -88.811 |
|  | 8 | 371.939 | 0.11953 | 4 | 0.998 | . | -88.9848 | -90.0564 | -88.8259 |
|  | 9 | 371.939 | 0 | 4 | . | . | -88.9848 | -90.0564 | -88.8259 |
|  | 10 | 371.939 | 0 | 4 | . | . | -88.9848* | -90.0564* | -88.8259* |
| India | 0 | -152.535 |  |  |  | 5.20E+16 | 44.1529 | 43.9619 | 44.1374 |
|  | 1 | -151.183 | 2.7044 | 4 | 0.608 | 1.20E+17 | 44.9094 | 44.3363 | 44.863 |
|  | 2 | -134.881 | 32.604 | 4 | 0 | 6.7e+15* | 41.3945 | 40.4395 | 41.3173 |
|  | 3 | 338.114 | 945.99 | 4 | 0 | . | -92.6041 | -93.9412 | -92.7123 |
|  | 4 | 345.506 | 14.784* | 4 | 0.005 | . | -94.7161 | -96.0532 | -94.8243 |
|  | 5 | 347.233 | 3.4543 | 4 | 0.485 | . | -95.2095* | -96.5466* | -95.3177* |
|  | 6 | 339.814 | -14.838 | 4 | . | . | -93.0898 | -94.4269 | -93.198 |
|  | 7 | 342.026 | 4.4233 | 4 | 0.352 | . | -93.7217 | -95.0588 | -93.8299 |
|  | 8 | 342.026 | 0 | 4 | . | . | -93.7217 | -95.0588 | -93.8299 |
|  | 9 | 342.026 | 0 | 4 | . | . | -93.7217 | -95.0588 | -93.8299 |
|  | 10 | 342.026 | 0 | 4 | . | . | -93.7217 | -95.0588 | -93.8299 |
| Indonesia | 0 | -169.326 |  |  |  | 1.40E+16 | 42.8315 | 42.6975 | 42.8513 |
|  | 1 | -167.096 | 4.4598 | 4 | 0.347 | 2.30E+16 | 43.274 | 42.8721 | 43.3336 |
|  | 2 | -162.097 | 9.9987 | 4 | 0.04 | 2.60E+16 | 43.0241 | 42.3544 | 43.1235 |
|  | 3 | . | . | 4 | . | 0* | . | . | . |
|  | 4 | 398.293 | . | 4 | . | . | -95.5733 | -96.6449 | -95.4144 |
|  | 5 | 398.293 | 0 | 4 | . | . | -95.5733 | -96.6449 | -95.4144 |
|  | 6 | 394.759 | -7.0689 | 4 | . | . | -94.6897 | -95.7613 | -94.5308 |
|  | 7 | 402.269 | 15.021* | 4 | 0.005 | . | -96.5673 | -97.6389 | -96.4084 |
|  | 8 | 405.025 | 5.511 | 4 | 0.239 | . | -97.2562 | -98.3278 | -97.0973 |
|  | 9 | 405.025 | 0 | 4 | . | . | -97.2562 | -98.3278 | -97.0973 |
|  | 10 | 405.025 | 0 | 4 | . | . | -97.2562* | -98.3278* | -97.0973* |
| Israel | 0 | -156.971 |  |  |  | 6.30E+14 | 39.7428 | 39.6089 | 39.7627 |
|  | 1 | -152.397 | 9.1489 | 4 | 0.057 | 5.80E+14 | 39.5992 | 39.1973 | 39.6588 |
|  | 2 | -146.739 | 11.316 | 4 | 0.023 | 5.50E+14 | 39.1846 | 38.5149 | 39.2839 |
|  | 3 | 9.74779 | 312.97 | 4 | 0 | .067434* | 1.06305 | 0.1254 | 1.20208 |
|  | 4 | 414.803 | 810.11 | 4 | 0 | . | -99.7007 | -100.772 | -99.5418 |
|  | 5 | 419.451 | 9.2972 | 4 | 0.054 | . | -100.863* | -101.934* | -100.704* |
|  | 6 | 407.315 | -24.272 | 4 | . | . | -97.8288 | -98.9004 | -97.6699 |
|  | 7 | 406.296 | -2.0392 | 4 | . | . | -97.5739 | -98.6455 | -97.415 |
|  | 8 | 414.887 | 17.182* | 4 | 0.002 | . | -99.7217 | -100.793 | -99.5628 |
|  | 9 | 411.132 | -7.509 | 4 | . | . | -98.7831 | -99.8547 | -98.6242 |
|  | 10 | 414.063 | 5.861 | 4 | 0.21 | . | -99.5157 | -100.587 | -99.3568 |
| Japan | 0 | -186.514 |  |  |  | 1.00E+18 | 47.1285 | 46.9945 | 47.1483 |
|  | 1 | -183.944 | 5.1399 | 4 | 0.273 | 1.60E+18 | 47.486 | 47.0841 | 47.5456 |
|  | 2 | -179.718 | 8.4516 | 4 | 0.076 | 2.10E+18 | 47.4295 | 46.7598 | 47.5288 |
|  | 3 | 12.1716 | 383.78 | 4 | 0 | .036789* | 0.457105 | -0.48055 | 0.596128 |
|  | 4 | 382.013 | 739.68 | 4 | 0 | . | -91.5034* | -92.575* | -91.3445* |
|  | 5 | 375.48 | -13.066 | 4 | . | . | -89.8701 | -90.9417 | -89.7112 |
|  | 6 | 368.612 | -13.738 | 4 | . | . | -88.1529 | -89.2245 | -87.994 |
|  | 7 | 376.181 | 15.138* | 4 | 0.004 | . | -90.0452 | -91.1168 | -89.8863 |
|  | 8 | 374.018 | -4.3255 | 4 | . | . | -89.5045 | -90.5761 | -89.3456 |
|  | 9 | 365.232 | -17.572 | 4 | . | . | -87.3079 | -88.3795 | -87.1491 |
|  | 10 | 365.232 | 0 | 4 | . | . | -87.3079 | -88.3795 | -87.1491 |
| Kazakhstan | 0 | -163.273 |  |  |  | 3.00E+15 | 41.3183 | 41.1843 | 41.3381 |
|  | 1 | -150.422 | 25.703 | 4 | 0 | 3.60E+14 | 39.1054 | 38.7035 | 39.165 |
|  | 2 | -149.298 | 2.248 | 4 | 0.69 | 1.00E+15 | 39.8244 | 39.1546 | 39.9237 |
|  | 3 | 0.47142 | 299.54 | 4 | 0 | .685567* | 3.38215 | 2.44449 | 3.52117 |
|  | 4 | 397.351 | 793.76 | 4 | 0 | . | -95.3377 | -96.4093 | -95.1788 |
|  | 5 | 404.793 | 14.883 | 4 | 0.005 | . | -97.1982* | -98.2698* | -97.0393* |
|  | 6 | 400.385 | -8.8144 | 4 | . | . | -96.0964 | -97.168 | -95.9375 |
|  | 7 | 400.907 | 1.0432 | 4 | 0.903 | . | -96.2268 | -97.2984 | -96.0679 |
|  | 8 | 391.552 | -18.71 | 4 | . | . | -93.888 | -94.9596 | -93.7291 |
|  | 9 | 403.504 | 23.905* | 4 | 0 | . | -96.8761 | -97.9477 | -96.7172 |
|  | 10 | 399.318 | -8.3736 | 4 | . | . | -95.8294 | -96.901 | -95.6705 |
| Korea Republic | 0 | -166.388 |  |  |  | 6.60E+15 | 42.097 | 41.9631 | 42.1169 |
|  | 1 | -166.172 | 0.43261 | 4 | 0.98 | 1.80E+16 | 43.0429 | 42.6411 | 43.1025 |
|  | 2 | -131.086 | 70.172 | 4 | 0 | 1.10E+13 | 35.2715 | 34.6017 | 35.3708 |
|  | 3 | . | . | 4 | . | -.946488* | . | . | . |
|  | 4 | 362.412 | . | 4 | . | . | -86.603 | -87.6746 | -86.4441 |
|  | 5 | 387.169 | 49.514 | 4 | 0 | . | -92.7922 | -93.8638 | -92.6334 |
|  | 6 | 384.063 | -6.2111 | 4 | . | . | -92.0159 | -93.0875 | -91.857 |
|  | 7 | 389.049 | 9.9711 | 4 | 0.041 | . | -93.2622 | -94.3339 | -93.1034 |
|  | 8 | 389.049 | 0 | 4 | . | . | -93.2622 | -94.3339 | -93.1034 |
|  | 9 | 387.04 | -4.018 | 4 | . | . | -92.76 | -93.8316 | -92.6011 |
|  | 10 | 397.423 | 20.765* | 4 | 0 | . | -95.3557* | -96.4273* | -95.1968* |
| Kuwait | 0 | -152.282 |  |  |  | 1.90E+14 | 38.5704 | 38.4364 | 38.5902 |
|  | 1 | -150.504 | 3.5547 | 4 | 0.47 | 3.60E+14 | 39.126 | 38.7242 | 39.1856 |
|  | 2 | -145.643 | 9.7215 | 4 | 0.045 | 4.20E+14 | 38.9109 | 38.2411 | 39.0102 |
|  | 3 | 9.4455 | 310.18 | 4 | 0 | .072728* | 1.13863 | 0.200974 | 1.27765 |
|  | 4 | 404.106 | 789.32 | 4 | 0 | . | -97.0265 | -98.0982 | -96.8677 |
|  | 5 | 411.428 | 14.644 | 4 | 0.005 | . | -98.857* | -99.9286* | -98.6981* |
|  | 6 | 408.885 | -5.086 | 4 | . | . | -98.2213 | -99.2929 | -98.0624 |
|  | 7 | 396.613 | -24.544 | 4 | . | . | -95.1532 | -96.2248 | -94.9944 |
|  | 8 | 411.406 | 29.586* | 4 | 0 | . | -98.8514 | -99.923 | -98.6926 |
|  | 9 | 411.406 | 0 | 4 | . | . | -98.8514 | -99.923 | -98.6926 |
|  | 10 | 411.406 | 0 | 4 | . | . | -98.8514 | -99.923 | -98.6926 |
| Kyrgyz Republic | 0 | -121.406 |  |  |  | 8.60E+10 | 30.8515 | 30.7176 | 30.8714 |
|  | 1 | -119.656 | 3.5007 | 4 | 0.478 | 1.60E+11 | 31.4139 | 31.0121 | 31.4735 |
|  | 2 | -118.021 | 3.2686 | 4 | 0.514 | 4.20E+11 | 32.0054 | 31.3356 | 32.1047 |
|  | 3 | 43.4792 | 323 | 4 | 0 | .000015* | -7.3698 | -8.30745 | -7.23078 |
|  | 4 | 431.761 | 776.56 | 4 | 0 | . | -103.94 | -105.012 | -103.781 |
|  | 5 | 436.764 | 10.007 | 4 | 0.04 | . | -105.191 | -106.263 | -105.032 |
|  | 6 | 434.574 | -4.3798 | 4 | . | . | -104.644 | -105.715 | -104.485 |
|  | 7 | 433.271 | -2.6056 | 4 | . | . | -104.318 | -105.389 | -104.159 |
|  | 8 | 438.719 | 10.894* | 4 | 0.028 | . | -105.68 | -106.751 | -105.521 |
|  | 9 | 438.719 | 0 | 4 | . | . | -105.68* | -106.751* | -105.521* |
|  | 10 | 431.329 | -14.779 | 4 | . | . | -103.832 | -104.904 | -103.673 |
| Malaysia | 0 | -152.693 |  |  |  | 2.10E+14 | 38.6733 | 38.5394 | 38.6932 |
|  | 1 | -149.516 | 6.3538 | 4 | 0.174 | 2.80E+14 | 38.8791 | 38.4772 | 38.9387 |
|  | 2 | -148.326 | 2.3812 | 4 | 0.666 | 8.20E+14 | 39.5814 | 38.9117 | 39.6807 |
|  | 3 | 20.854 | 338.36 | 4 | 0 | .004198* | -1.71351 | -2.65116 | -1.57449 |
|  | 4 | 375.162 | 708.62 | 4 | 0 | . | -89.7905 | -90.8621 | -89.6316 |
|  | 5 | 394.038 | 37.751 | 4 | 0 | . | -94.5094 | -95.581 | -94.3505 |
|  | 6 | 393.627 | -0.82159 | 4 | . | . | -94.4067 | -95.4783 | -94.2478 |
|  | 7 | 400.355 | 13.456 | 4 | 0.009 | . | -96.0887 | -97.1603 | -95.9298 |
|  | 8 | 405.169 | 9.6289 | 4 | 0.047 | . | -97.2923 | -98.3639 | -97.1334 |
|  | 9 | 398.406 | -13.526 | 4 | . | . | -95.6015 | -96.6731 | -95.4426 |
|  | 10 | 410.104 | 23.396* | 4 | 0 | . | -98.526* | -99.5976* | -98.3671* |
| Maldives | 0 | -108.866 |  |  |  | 3.70E+09 | 27.7164 | 27.5825 | 27.7363 |
|  | 1 | -102.128 | 13.476 | 4 | 0.009 | 2.00E+09 | 27.0319 | 26.63 | 27.0915 |
|  | 2 | -100.943 | 2.3692 | 4 | 0.668 | 5.90E+09 | 27.7357 | 27.066 | 27.835 |
|  | 3 | . | . | 4 | . | 0* | . | . | . |
|  | 4 | 431.417 | . | 4 | . | . | -103.854 | -104.926 | -103.695 |
|  | 5 | 456.033 | 49.232 | 4 | 0 | . | -110.008 | -111.08 | -109.849 |
|  | 6 | 454.398 | -3.2708 | 4 | . | . | -109.599 | -110.671 | -109.441 |
|  | 7 | 456.154 | 3.5119 | 4 | 0.476 | . | -110.038 | -111.11 | -109.88 |
|  | 8 | 466.85 | 21.392 | 4 | 0 | . | -112.712* | -113.784* | -112.554* |
|  | 9 | 455.379 | -22.942 | 4 | . | . | -109.845 | -110.916 | -109.686 |
|  | 10 | 461.318 | 11.879* | 4 | 0.018 | . | -111.33 | -112.401 | -111.171 |
| Mongolia | 0 | -141.295 |  |  |  | 1.20E+13 | 35.8238 | 35.6898 | 35.8436 |
|  | 1 | -138.128 | 6.3333 | 4 | 0.176 | 1.60E+13 | 36.0321 | 35.6302 | 36.0917 |
|  | 2 | -135.982 | 4.2924 | 4 | 0.368 | 3.70E+13 | 36.4955 | 35.8258 | 36.5948 |
|  | 3 | 27.9572 | 327.88 | 4 | 0 | .000711* | -3.48931 | -4.42696 | -3.35029 |
|  | 4 | 404.666 | 753.42 | 4 | 0 | . | -97.1664 | -98.238 | -97.0075 |
|  | 5 | 413.732 | 18.133 | 4 | 0.001 | . | -99.4331 | -100.505 | -99.2742 |
|  | 6 | 428.425 | 29.386* | 4 | 0 | . | -103.106 | -104.178 | -102.947 |
|  | 7 | 428.425 | 0 | 4 | . | . | -103.106 | -104.178 | -102.947 |
|  | 8 | 428.425 | 0 | 4 | . | . | -103.106 | -104.178 | -102.947 |
|  | 9 | 428.425 | 0 | 4 | . | . | -103.106 | -104.178 | -102.947 |
|  | 10 | 428.425 | 0 | 4 | . | . | -103.106* | -104.178* | -102.947* |
| Nepal | 0 | -116.357 |  |  |  | 2.40E+10 | 29.5893 | 29.4553 | 29.6091 |
|  | 1 | -114.002 | 4.71 | 4 | 0.318 | 4.00E+10 | 30.0005 | 29.5987 | 30.0601 |
|  | 2 | -104.745 | 18.515 | 4 | 0.001 | 1.50E+10 | 28.6862 | 28.0165 | 28.7855 |
|  | 3 | . | . | 4 | . | -1.2e-07* | . | . | . |
|  | 4 | 422.03 | . | 4 | . | . | -101.507 | -102.579 | -101.349 |
|  | 5 | 428.065 | 12.071 | 4 | 0.017 | . | -103.016 | -104.088 | -102.857 |
|  | 6 | 448.016 | 39.901* | 4 | 0 | . | -108.004 | -109.076 | -107.845 |
|  | 7 | 449.433 | 2.8338 | 4 | 0.586 | . | -108.358 | -109.43 | -108.199 |
|  | 8 | 449.433 | 0 | 4 | . | . | -108.358 | -109.43 | -108.199 |
|  | 9 | 449.433 | 0 | 4 | . | . | -108.358 | -109.43 | -108.199 |
|  | 10 | 449.433 | 0 | 4 | . | . | -108.358* | -109.43* | -108.199* |
| Oman | 0 | -153.456 |  |  |  | 2.60E+14 | 38.8639 | 38.73 | 38.8838 |
|  | 1 | -146.015 | 14.882 | 4 | 0.005 | 1.20E+14 | 38.0036 | 37.6018 | 38.0632 |
|  | 2 | -136.441 | 19.148 | 4 | 0.001 | 4.20E+13 | 36.6102 | 35.9404 | 36.7095 |
|  | 3 | . | . | 4 | . | 0* | . | . | . |
|  | 4 | 395.156 | . | 4 | . | . | -94.7891 | -95.8607 | -94.6302 |
|  | 5 | 411.049 | 31.785* | 4 | 0 | . | -98.7622* | -99.8338* | -98.6033* |
|  | 6 | 409.688 | -2.7224 | 4 | . | . | -98.4219 | -99.4935 | -98.263 |
|  | 7 | 409.688 | 0 | 4 | . | . | -98.4219 | -99.4935 | -98.263 |
|  | 8 | 409.688 | 0 | 4 | . | . | -98.4219 | -99.4935 | -98.263 |
|  | 9 | 403.528 | -12.318 | 4 | . | . | -96.8821 | -97.9537 | -96.7232 |
|  | 10 | 400.452 | -6.1527 | 4 | . | . | -96.113 | -97.1846 | -95.9542 |
| Pakistan | 0 | -141.441 |  |  |  | 1.30E+13 | 35.8603 | 35.7264 | 35.8802 |
|  | 1 | -137.251 | 8.3807 | 4 | 0.079 | 1.30E+13 | 35.8128 | 35.4109 | 35.8723 |
|  | 2 | -128.189 | 18.124 | 4 | 0.001 | 5.30E+12 | 34.5473 | 33.8776 | 34.6466 |
|  | 3 | 17.3437 | 291.07 | 4 | 0 | .010096* | -0.83593 | -1.77358 | -0.6969 |
|  | 4 | 393.933 | 753.18 | 4 | 0 | . | -94.4833 | -95.5549 | -94.3244 |
|  | 5 | 394.535 | 1.2029 | 4 | 0.878 | . | -94.6337 | -95.7053 | -94.4748 |
|  | 6 | 402.007 | 14.944 | 4 | 0.005 | . | -96.5016 | -97.5732 | -96.3428 |
|  | 7 | 411.991 | 19.968 | 4 | 0.001 | . | -98.9977 | -100.069 | -98.8388 |
|  | 8 | 431.707 | 39.432* | 4 | 0 | . | -103.927 | -104.998 | -103.768 |
|  | 9 | 428.61 | -6.1949 | 4 | . | . | -103.152 | -104.224 | -102.994 |
|  | 10 | 432.921 | 8.6229 | 4 | 0.071 | . | -104.23* | -105.302* | -104.071* |
| Philippines | 0 | -132.749 |  |  |  | 1.8e+14* | 38.4996 | 38.3086 | 38.4842 |
|  | 1 | -131.006 | 3.4848 | 4 | 0.48 | 3.90E+14 | 39.1447 | 38.5716 | 39.0983 |
|  | 2 | -128.103 | 5.8063 | 4 | 0.214 | 9.70E+14 | 39.458 | 38.503 | 39.3808 |
|  | 3 | 350.22 | 956.65 | 4 | 0 | . | -96.0629 | -97.4 | -96.1711 |
|  | 4 | 350.298 | 0.15516 | 4 | 0.997 | . | -96.0851 | -97.4221 | -96.1932 |
|  | 5 | 344.16 | -12.275 | 4 | . | . | -94.3314 | -95.6685 | -94.4396 |
|  | 6 | 357.292 | 26.264 | 4 | 0 | . | -98.0834 | -99.4205 | -98.1916 |
|  | 7 | 362.832 | 11.081 | 4 | 0.026 | . | -99.6664 | -101.003 | -99.7745 |
|  | 8 | 367.902 | 10.14* | 4 | 0.038 | . | -101.115* | -102.452* | -101.223* |
|  | 9 | 357.761 | -20.282 | 4 | . | . | -98.2175 | -99.5546 | -98.3257 |
|  | 10 | 361.774 | 8.0246 | 4 | 0.091 | . | -99.3639 | -100.701 | -99.4721 |
| Saudi Arabia | 0 | -155.526 |  |  |  | 1.2e+17* | 45.0075 | 44.8165 | 44.9921 |
|  | 1 | -153.114 | 4.8257 | 4 | 0.306 | 2.10E+17 | 45.461 | 44.888 | 45.4146 |
|  | 2 | -148.349 | 9.5298 | 4 | 0.049 | 3.20E+17 | 45.2425 | 44.2874 | 45.1652 |
|  | 3 | 301.956 | 900.61 | 4 | 0 | . | -82.273 | -83.6101 | -82.3812 |
|  | 4 | 307.105 | 10.298 | 4 | 0.036 | . | -83.7442 | -85.0813 | -83.8524 |
|  | 5 | 341.125 | 68.04 | 4 | 0 | . | -93.4642* | -94.8013* | -93.5724* |
|  | 6 | 325.814 | -30.621 | 4 | . | . | -89.0898 | -90.4268 | -89.1979 |
|  | 7 | 339.403 | 27.177* | 4 | 0 | . | -92.9722 | -94.3093 | -93.0804 |
|  | 8 | 335.106 | -8.5936 | 4 | . | . | -91.7446 | -93.0816 | -91.8527 |
|  | 9 | 336.571 | 2.93 | 4 | 0.57 | . | -92.1631 | -93.5002 | -92.2713 |
|  | 10 | 336.571 | 0 | 4 | . | . | -92.1631 | -93.5002 | -92.2713 |
| Singapore | 0 | -172.327 |  |  |  | 2.90E+16 | 43.5816 | 43.4477 | 43.6015 |
|  | 1 | -163.129 | 18.394 | 4 | 0.001 | 8.50E+15 | 42.2824 | 41.8805 | 42.3419 |
|  | 2 | -160.058 | 6.1431 | 4 | 0.189 | 1.50E+16 | 42.5145 | 41.8447 | 42.6138 |
|  | 3 | . | . | 4 | . | -5.48772* | . | . | . |
|  | 4 | 379.124 | . | 4 | . | . | -90.7809 | -91.8525 | -90.622 |
|  | 5 | 387.672 | 17.098 | 4 | 0.002 | . | -92.9181 | -93.9897 | -92.7592 |
|  | 6 | 385.21 | -4.9241 | 4 | . | . | -92.3026 | -93.3742 | -92.1437 |
|  | 7 | 383.896 | -2.6295 | 4 | . | . | -91.9739 | -93.0455 | -91.815 |
|  | 8 | 382.461 | -2.8695 | 4 | . | . | -91.6152 | -92.6868 | -91.4563 |
|  | 9 | 388.644 | 12.366 | 4 | 0.015 | . | -93.161 | -94.2326 | -93.0021 |
|  | 10 | 397.049 | 16.81* | 4 | 0.002 | . | -95.2623* | -96.3339* | -95.1034* |
| Solomon Islands | 0 | -73.9133 |  |  |  | 600306 | 18.9783 | 18.8444 | 18.9982 |
|  | 1 | -67.0368 | 13.753 | 4 | 0.008 | 315011 | 18.2592 | 17.8574 | 18.3188 |
|  | 2 | -55.5201 | 23.033 | 4 | 0 | 68662.2 | 16.38 | 15.7103 | 16.4793 |
|  | 3 | . | . | 4 | . | -2.4e-10* | . | . | . |
|  | 4 | 469.287 | . | 4 | . | . | -113.322 | -114.393 | -113.163 |
|  | 5 | 469.013 | -0.54862 | 4 | . | . | -113.253 | -114.325 | -113.094 |
|  | 6 | 483.33 | 28.634 | 4 | 0 | . | -116.832 | -117.904 | -116.674 |
|  | 7 | 470.53 | -25.6 | 4 | . | . | -113.632 | -114.704 | -113.474 |
|  | 8 | 493.324 | 45.589* | 4 | 0 | . | -119.331 | -120.403 | -119.172 |
|  | 9 | 493.324 | 0 | 4 | . | . | -119.331 | -120.403 | -119.172 |
|  | 10 | 493.324 | 0 | 4 | . | . | -119.331* | -120.403* | -119.172* |
| Sri Lanka | 0 | -121.352 |  |  |  | 8.50E+10 | 30.8381 | 30.7042 | 30.858 |
|  | 1 | -120.762 | 1.1813 | 4 | 0.881 | 2.10E+11 | 31.6904 | 31.2886 | 31.75 |
|  | 2 | -104.207 | 33.109 | 4 | 0 | 1.30E+10 | 28.5518 | 27.882 | 28.6511 |
|  | 3 | 42.242 | 292.9 | 4 | 0 | .00002* | -7.06051 | -7.99816 | -6.92149 |
|  | 4 | 419.178 | 753.87 | 4 | 0 | . | -100.795 | -101.866 | -100.636 |
|  | 5 | 416.659 | -5.0384 | 4 | . | . | -100.165 | -101.236 | -100.006 |
|  | 6 | 411.279 | -10.76 | 4 | . | . | -98.8198 | -99.8914 | -98.6609 |
|  | 7 | 398.96 | -24.639 | 4 | . | . | -95.7399 | -96.8115 | -95.581 |
|  | 8 | 434.253 | 70.587 | 4 | 0 | . | -104.563 | -105.635 | -104.404 |
|  | 9 | 432.866 | -2.7742 | 4 | . | . | -104.217 | -105.288 | -104.058 |
|  | 10 | 447.502 | 29.271* | 4 | 0 | . | -107.875* | -108.947* | -107.717* |
| Thailand | 0 | -168.189 |  |  |  | 1.00E+16 | 42.5473 | 42.4133 | 42.5671 |
|  | 1 | -164.289 | 7.8003 | 4 | 0.099 | 1.10E+16 | 42.5722 | 42.1704 | 42.6318 |
|  | 2 | -161.582 | 5.4147 | 4 | 0.247 | 2.30E+16 | 42.8954 | 42.2257 | 42.9947 |
|  | 3 | . | . | 4 | . | -.174724* | . | . | . |
|  | 4 | 374.293 | . | 4 | . | . | -89.5733 | -90.6449 | -89.4144 |
|  | 5 | 390.568 | 32.551 | 4 | 0 | . | -93.6421 | -94.7137 | -93.4832 |
|  | 6 | 395.952 | 10.767* | 4 | 0.029 | . | -94.988 | -96.0596 | -94.8291 |
|  | 7 | 398.258 | 4.6112 | 4 | 0.33 | . | -95.5644* | -96.636* | -95.4055* |
|  | 8 | 389.717 | -17.082 | 4 | . | . | -93.4292 | -94.5008 | -93.2703 |
|  | 9 | 394.369 | 9.3047 | 4 | 0.054 | . | -94.5923 | -95.6639 | -94.4334 |
|  | 10 | 394.437 | 0.13588 | 4 | 0.998 | . | -94.6092 | -95.6808 | -94.4504 |
| Vanuatu | 0 | -79.1501 |  |  |  | 2.20E+06 | 20.2875 | 20.1536 | 20.3074 |
|  | 1 | -74.625 | 9.0502 | 4 | 0.06 | 2.10E+06 | 20.1563 | 19.7544 | 20.2158 |
|  | 2 | -73.0236 | 3.2029 | 4 | 0.524 | 5.50E+06 | 20.7559 | 20.0861 | 20.8552 |
|  | 3 | 73.6065 | 293.26 | 4 | 0 | 7.9e-09* | -14.9016 | -15.8393 | -14.7626 |
|  | 4 | 455.489 | 763.77 | 4 | 0 | . | -109.872 | -110.944 | -109.713 |
|  | 5 | 477.677 | 44.375 | 4 | 0 | . | -115.419 | -116.491 | -115.26 |
|  | 6 | 492.106 | 28.858* | 4 | 0 | . | -119.026* | -120.098* | -118.868* |
|  | 7 | 476.158 | -31.896 | 4 | . | . | -115.04 | -116.111 | -114.881 |
|  | 8 | 480.785 | 9.2534 | 4 | 0.055 | . | -116.196 | -117.268 | -116.037 |
|  | 9 | 473.918 | -13.733 | 4 | . | . | -114.48 | -115.551 | -114.321 |
|  | 10 | 477.863 | 7.8903 | 4 | 0.096 | . | -115.466 | -116.537 | -115.307 |
| Vietnam | 0 | -141.663 |  |  |  | 1.40E+13 | 35.9156 | 35.7817 | 35.9355 |
|  | 1 | -138.917 | 5.491 | 4 | 0.241 | 2.00E+13 | 36.2293 | 35.8274 | 36.2889 |
|  | 2 | -120.027 | 37.781 | 4 | 0 | 6.90E+11 | 32.5067 | 31.8369 | 32.606 |
|  | 3 | . | . | 4 | . | -.008062* | . | . | . |
|  | 4 | 424.213 | . | 4 | . | . | -102.053 | -103.125 | -101.894 |
|  | 5 | 416.844 | -14.74 | 4 | . | . | -100.211 | -101.282 | -100.052 |
|  | 6 | 416.957 | 0.22642 | 4 | 0.994 | . | -100.239 | -101.311 | -100.08 |
|  | 7 | 425.639 | 17.366 | 4 | 0.002 | . | -102.41 | -103.481 | -102.251 |
|  | 8 | 430.539 | 9.7991* | 4 | 0.044 | . | -103.635 | -104.706 | -103.476 |
|  | 9 | 430.539 | 0 | 4 | . | . | -103.635 | -104.706 | -103.476 |
|  | 10 | 430.539 | 0 | 4 | . | . | -103.635* | -104.706* | -103.476* |

## Table 06: PVAR lag length criteria

| **PVAR Lag Selection Criteria** | | | | | | |
| --- | --- | --- | --- | --- | --- | --- |
|  | Lag | J | J Pvalue | MBIC | MAIC | MQIC |
| All countries | 1 | 0.59086 | 44.4196 | 0.158354 | -182.738 | -27.5804 |
|  | 2 | 0.667538 | 41.65587 | 0.11804 | -160.262 | -22.3441 |
|  | 3 | 0.837397 | 39.48481 | 0.073434 | -137.193 | -16.5152 |
|  | 4 | 0.858654 | 34.69514 | 0.073051 | -116.743 | -13.3049 |
|  | 5 | 0.847343 | 37.42232 | 0.010409 | -88.7761 | -2.57768 |
|  | 6 | 0.902713 | 29.31872 | 0.021874 | -71.64 | -2.68128 |
|  | 7 | 0.923025 | 19.03009 | 0.087805 | -56.6889 | -4.96991 |
|  | 8 | 0.895446 | 15.4244 | 0.0514 | -35.0549 | -0.5756 |
|  | 9 | 0.960071 | 9.625702 | 0.047228 | -15.614 | 1.625702 |
| Africa | 1 | -0.49337 | 33.74628 | 0.576236 | -140.36 | -38.2537 |
|  | 2 | 0.430674 | 30.32026 | 0.551691 | -124.441 | -33.6797 |
|  | 3 | 0.706652 | 29.33191 | 0.395841 | -106.084 | -26.6681 |
|  | 4 | 0.82624 | 20.1324 | 0.689222 | -95.9384 | -27.8676 |
|  | 5 | 0.815025 | 20.92476 | 0.401573 | -75.8009 | -19.0752 |
|  | 6 | 0.80026 | 15.32957 | 0.500645 | -62.0509 | -16.6704 |
|  | 7 | 0.893878 | 17.22654 | 0.141272 | -40.8088 | -6.77346 |
|  | 8 | 0.939101 | 12.34829 | 0.136328 | -26.342 | -3.65171 |
|  | 9 | 0.946682 | 6.085708 | 0.192838 | -13.2594 | -1.91429 |
| Asia & Oceania | 1 | 0.181095 | 38.39485 | 0.361499 | -151.617 | -33.6052 |
|  | 2 | 0.441441 | 42.1485 | 0.108173 | -126.751 | -21.8515 |
|  | 3 | 0.587887 | 36.52981 | 0.129597 | -111.257 | -19.4702 |
|  | 4 | 0.728367 | 32.90321 | 0.106132 | -93.7715 | -15.0968 |
|  | 5 | 0.682325 | 32.12957 | 0.041937 | -73.4327 | -7.87043 |
|  | 6 | 0.838954 | 23.11021 | 0.11082 | -61.3396 | -8.88979 |
|  | 7 | 0.857934 | 14.92417 | 0.245609 | -48.4132 | -9.07583 |
|  | 8 | 0.870738 | 14.19817 | 0.076744 | -28.0268 | -1.80183 |
|  | 9 | 0.883622 | 8.505104 | 0.074733 | -12.6074 | 0.505104 |
| Europe | 1 | 0.273493 | 37.66995 | 0.392728 | -153.782 | -34.3301 |
|  | 2 | 0.320042 | 40.70117 | 0.13919 | -129.479 | -23.2988 |
|  | 3 | 0.525732 | 33.91062 | 0.203831 | -114.997 | -22.0894 |
|  | 4 | 0.41215 | 34.43238 | 0.077273 | -93.2025 | -13.5676 |
|  | 5 | 0.672217 | 25.40139 | 0.186504 | -80.961 | -14.5986 |
|  | 6 | 0.735201 | 18.57954 | 0.291068 | -66.5104 | -13.4205 |
|  | 7 | 0.812034 | 14.10199 | 0.294243 | -49.7155 | -9.89801 |
|  | 8 | 0.817584 | 14.23417 | 0.075862 | -28.3108 | -1.76583 |
|  | 9 | 0.893263 | 5.446583 | 0.244465 | -15.8259 | -2.55342 |
| North America | 1 | 0.988352 | 32.42494 | 0.639381 | -144.431 | -39.5751 |
|  | 2 | 0.994019 | 39.7868 | 0.162093 | -117.418 | -24.2132 |
|  | 3 | 0.998403 | 31.16938 | 0.309611 | -106.385 | -24.8306 |
|  | 4 | 0.999612 | 29.36871 | 0.206563 | -88.535 | -18.6313 |
|  | 5 | 0.999919 | 26.47057 | 0.150827 | -71.7825 | -13.5294 |
|  | 6 | 0.999986 | 16.66665 | 0.407479 | -61.9358 | -15.3334 |
|  | 7 | 0.999996 | 21.32331 | 0.045842 | -37.6286 | -2.67669 |
|  | 8 | 0.999989 | 10.7354 | 0.217147 | -28.5658 | -5.2646 |
|  | 9 | 0.999991 | 9.955669 | 0.041181 | -9.69495 | 1.955669 |
| South America | 1 | 0.691386 | 34.84936 | 0.523204 | -112.547 | -37.1506 |
|  | 2 | 0.750666 | 33.53902 | 0.392617 | -97.48 | -30.461 |
|  | 3 | 0.760305 | 33.67097 | 0.211917 | -80.9707 | -22.329 |
|  | 4 | 0.7498 | 27.8031 | 0.268433 | -70.4612 | -20.1969 |
|  | 5 | 0.844935 | 25.54954 | 0.18121 | -56.3374 | -14.4505 |
|  | 6 | 0.876028 | 22.69755 | 0.122062 | -42.812 | -9.30245 |
|  | 7 | 0.905645 | 21.73427 | 0.040608 | -27.3979 | -2.26573 |
|  | 8 | 0.948734 | 23.88661 | 0.002394 | -8.86815 | 7.886606 |
|  | 9 | 0.987004 | 11.56442 | 0.020902 | -4.81296 | 3.564419 |

Notes: MBIC = model/moment selection Bayesian information criterion; MAIC = model/moment selection Akaike information criterion; MQIC = model/moment selection Hannan and Quinn information criterion. * Denotes minimum values of MAIC, MBIC, MQIC.
