# Supplementary material for: From short to long term: Dynamic analysis of FDI and net export in global regions
Source: PLoS One. 2023 Sep 14;18(9):e0291301. doi: 10.1371/journal.pone.0291301 (PMC10501631; doi:10.1371/journal.pone.0291301)
Supplement: Attachment — Submitted filename: Response to the Reviewers.docx [file pone.0291301.s005.docx]

**Point by point response to reviewer 1**

Dear editor and the reviewer,

We would like to express our profound appreciation to you for the valuable comments and suggestions made on our manuscript which were very helpful in revising and improving it.

Please note that line numbers refereed in this document is align with the revised manuscript which has track changes document (attached after the following table).

| **Comments of Reviewer 1** | **Comments of Authors** |
| --- | --- |
| **1. Is the manuscript technically sound, and do the data support the conclusions?**  The manuscript must describe a technically sound piece of scientific research with data that supports the conclusions. Experiments must have been conducted rigorously, with appropriate controls, replication, and sample sizes. The conclusions must be drawn appropriately based on the data presented.  Reviewer #1: **No** | Well received & noted. The discussion and conclusion sections has been amended to considering the comments and is now aligned. |
| **2. Has the statistical analysis been performed appropriately and rigorously?**  Reviewer #1: **No** | Noted the comment. We have used the wavelet coherence analysis to |
| **3. Have the authors made all data underlying the findings in their manuscript fully available?**  The [PLOS Data policy](http://www.plosone.org/static/policies.action#sharing) requires authors to make all data underlying the findings described in their manuscript fully available without restriction, with rare exception (please refer to the Data Availability Statement in the manuscript PDF file). The data should be provided as part of the manuscript or its supporting information or deposited to a public repository. For example, in addition to summary statistics, the data points behind means, medians and variance measures should be available. If there are restrictions on publicly sharing data—e.g., participant privacy or use of data from a third party—those must be specified.  Reviewer #1: **Yes** | Sincere appreciate for the feedback and thank you. |
| **4. Is the manuscript presented in an intelligible fashion and written in standard English?**  PLOS ONE does not copyedit accepted manuscripts, so the language in submitted articles must be clear, correct, and unambiguous. Any typographical or grammatical errors should be corrected at revision, so please note any specific errors here.  Reviewer #1: **Yes** | Thank you. Comment has been noted. |
| **5. Review Comments to the Author** |  |
| 1. The equations are not described adequately. There is so missing information. I.e., what is the function φ in (1), how the parameters are estimated in (2), what is the function W(.) in (3) and so on. | Very much appreciated for your comment and equations have been described adequately, from 346 to 366.  “A wavelet, which was built by the function $\psi^{a,b}(x)$ ($\psi$ denotes wavelet function), with constructions, $a$ and translation, $b$ can be represented as follows,  $\psi^{a,b}\left( x \right)=\left\vert a \right\vert^{-\frac{1}{2}}\psi\left( \frac{x-b}{a} \right) \ldots\ldots.(1)$  Wavelet coherence can be considered as an augmentation of the Wavelet analysis. Wavelet coherence explicit how one variable leads to another in bivariate analysis. Wavelet coherence is superior to other analysis such as granger causality because of the ability that can explain intensity and direction of the relationship between two variables in different time period. With the investigation of two time series, Wavelet coherence is ablet to explain the direction from one variable to another. The wavelet coherence formula (Galappaththi et al., 2023), including smoothing factor, s, first variable, y and second variable x, is denoted as follows,  $Coherence=\frac{\left\vert sWave.xy \right\vert^{2}}{sPower.x\cdot sPower.y} \ldots.(2)$  The study considered Net export as the first variable, and the second variable was the Foreign direct investment. The data set that used in the study is attached in S2 appendix and Wavelet coherence graphs were illustrated using R software.” |
| 2. In (7) the null hypothesis would have tested for a parameter named δ, but such a parameter does not exist in equation (7). | Thank you. Comment has been noted. The methodology section has been rewritten; the equation number changed from equation (7) to equation (3) The parameter already exists in equation (3). Equation (3) has been described in line from 368 to 377.  “There are different approaches to testing causality in panel data series. This study used cross-country VAR Granger analysis (Galappaththi et al., 2023), to evaluate the causality between FDI and NE for each country undertaken in the study. Initially, the study ran the Augmented Dickey–Fuller test (ADF/dfuller) (Dickey & Fuller, 1979) and Phillips–Perron test (PP) (Phillips & Perron, 1988) to assess the stationary$\left( H_{0}: \delta=0 \right)$ and non-stationary$\left( H_{A}: \delta\neq0 \right)$ of the two-time series (Hameed, Rahman, & Khanam, 2022). Thus, ADF Equation is as below.  $\Delta y_{t}=\alpha+\beta t+\gamma y_{t-1}+\delta_{1}\Delta y_{t-1}+\cdots\delta_{p-1}\Delta y_{t-P+}\varepsilon_{t}, \ldots\ldots\ldots(3)$  ADF (dfuller) test results in a negative number; when the number is more negative, the hypothesis is rejected that there is a unit root with a high level of confidence. Here, $\alpha$ denotes the constant of the time series. $\beta$ indicates the coefficient of the time series, and $\rho$ prescribes the lag order. Similarly, the PP test is derived from the dfuller test.” |
| 3. Overall, the authors have been confused with the illustration of the equations. | Thank you for the comment. We have re-illustrate the equations clearly in line from 346 to 366 and from 378 to 388.  “A wavelet, which was built by the function $\psi^{a,b}(x)$ ($\psi$ denotes wavelet function), with constructions, $a$ and translation, $b$ can be represented as follows,  $\psi^{a,b}\left( x \right)=\left\vert a \right\vert^{-\frac{1}{2}}\psi\left( \frac{x-b}{a} \right) \ldots\ldots.(1)$  Wavelet coherence can be considered as an augmentation of the Wavelet analysis. Wavelet coherence explicit how one variable leads to another in bivariate analysis. Wavelet coherence is superior to other analysis such as granger causality because of the ability that can explain intensity and direction of the relationship between two variables in different time period. With the investigation of two time series, Wavelet coherence is ablet to explain the direction from one variable to another. The wavelet coherence formula (Galappaththi et al., 2023), including smoothing factor, s, first variable, y and second variable x, is denoted as follows,  $Coherence=\frac{\left\vert sWave.xy \right\vert^{2}}{sPower.x\cdot sPower.y} \ldots.(2)$  The study considered Net export as the first variable, and the second variable was the Foreign direct investment. The data set that used in the study is attached in S2 appendix and Wavelet coherence graphs were illustrated using R software.”  “The Granger Causality runs along each individual country to further enhancement of the result that obtained from Wavelet Coherence technique. Here, $X$ and $Y$ are the two stationary covariance variables. The variable $X_{i't}$ causes $Y_{i,t}$, if we are better able to predict $Y_{i,t}$ by utilizing all variable information, compared to the use of information except $X_{i,t}$ for each individual $i \epsilon[1,N]$. The test assumed that the model is linear, thus use time stationary VAR representation for a data set to each cross-section unit $i$ and time period $t$, considering those aspect the equation 8 is estimated,  $Y_{i,t}= \sum_{k=1}^{p} \beta_{k}Y_{i,t-k}+ \sum_{k=0}^{p} \theta_{k}X_{i,t-k}+u_{i,t} \ldots\ldots\ldots\ldots\ldots\ldots\ldots\ldots\ldots\ldots\ldots\ldots\ldots(3)$  Here, $u$ is normally distributed with $u_{i,t}= \alpha_{i}+ \varepsilon_{i,t},P$ represents the number of lags. The equation assumed that $\beta_{k}$ is the auto regressive coefficients and $\theta_{k}$ is the regression coefficients. Those coefficients are constant for $k \epsilon[1,N]$ (Galappaththi et al., 2023).” |
| 4. There are too many grammatical (apart from the mathematical) errors. |  |
| 5. One more issue that is important is the usability of the papers’ estimations. Even if the presentation of the equation was fine, definitely, the Wavelet analysis and the causality tests (as they were presented) do not assist governments and policymakers to their policies with FDI flows. | Thank you. Comment has been noted and policy implications has been added in line from 711 to 729.  “The findings of this research have important implications for global institutions and policy-making bodies, such as governments, as they seek to understand the implications of policies related to exports and foreign direct investment (FDI) over the past two decades. By learning from the patterns observed in this study, policymakers can develop more effective policies that will help them achieve their intended objectives.  While most of countries have shown a positive bidirectional causality between exports and FDI, it is important to note that the American and African regions have experienced a negative impact of FDI on net exports.  This suggests that policies that aim to attract FDI in these regions (American and African regions) should be carefully designed to avoid negative effects on net exports. To achieve this, policymakers should focus on creating an enabling environment that supports export-oriented industries and promotes domestic production. This could involve implementing policies that provide incentives for companies to produce locally, reducing trade barriers, and investing in infrastructure and technology to improve productivity and competitiveness. Additionally, flexible trade policies, favourable tax conditions for exporters, and an accessible market for foreign investors may help countries in these regions attract more FDI without negatively impacting net exports. As multinational companies play a crucial role in both FDI and trade, policymakers should prioritize the easing of trade barriers and the development of trade facilities that can help countries attract more investment while ensuring that the benefits of FDI are not outweighed by negative impacts on net exports.  Overall, these policy implications highlight the importance of creating an environment that fosters international trade and investment while also promoting domestic production and competitiveness. By taking steps to strike a balance between FDI and net exports, governments can boost economic growth and promote long-term development.” |
